# Development and validation of a prediction model for VTE risk in gastric and esophageal cancer patients

**DOI:** 10.3389/fphar.2025.1448879

**Published:** 2025-02-28

**Authors:** Xingyue Zheng, Liuyun Wu, Lian Li, Yin Wang, Qinan Yin, Lizhu Han, Xingwei Wu, Yuan Bian

**Affiliations:** Department of Pharmacy, Personalized Drug Therapy Key Laboratory of Sichuan Province, Sichuan Academy of Medical Sciences and Sichuan Provincial People’s Hospital, School of Medicine, University of Electronic Science and Technology of China, Chengdu, China

**Keywords:** Gastric cancer, Esophageal cancer, venous thromboembolism, risk factors, prediction model

## Abstract

**Objective:**

This study focuses on the risk of venous thromboembolism (VTE) in patients with gastric or esophageal cancer (GC/EC), investigating the risk factors for VTE in this population. Utilizing machine learning techniques, the research aims to develop an interpretable VTE risk prediction model. The goal is to identify patients with gastric or esophageal cancer who are at high risk of VTE at an early stage in clinical practice, thereby enabling precise anticoagulant prophylaxis and thrombus management.

**Methods:**

This study is a real-world investigation aimed at predicting VTE in patients with GC/EC. Data were collected from inpatients diagnosed with GC/EC at Sichuan Provincial People’s Hospital between 1 January 2018, and 31 June 2023. Using nine supervised learning algorithms, 576 prediction models were developed based on 56 available variables. Subsequently, a simplified modeling approach was employed using the top 12 feature variables from the best-performing model. The primary metric for assessing the predictive performance of the models was the area under the ROC curve (AUC). Additionally, the training data used to construct the best model in this study were employed to externally validate several existing assessment models, including the Padua, Caprini, Khorana, and COMPASS-CAT scores.

**Results:**

A total of 3,742 cases of GC/EC patients were collected after excluding duplicate visit information. The study included 861 (23.0%) patients, of which 124 (14.4%) developed VTE. The top five models based on AUC for full-variable modeling are as follows: GBoost (0.9646), Logic Regression (0.9443), AdaBoost (0.9382), CatBoost (0.9354), XGBoost (0.8097). For simplified modeling, the models are: Simp-CatBoost (0.8811), Simp-GBoost (0.8771), Simp-Random Forest (0.8736), Simp-AdaBoost (0.8263), Simp-Logistic Regression (0.8090). After evaluating predictive performance and practicality, the Simp-GBoost model was determined as the best model for this study. External validation of the Padua score, Caprini score, Khorana score, and COMPASS-CAT score based on the training set of the Simp-GBoost model yielded AUCs of 0.4367, 0.2900, 0.5000, and 0.3633, respectively.

**Conclusion:**

In this study, we analyzed the risk factors of VTE in GC/EC patients, and constructed a well-performing VTE risk prediction model capable of accurately identifying the extent of VTE risk in patients. Four VTE prediction scoring systems were introduced to externally validate the dataset of this study. The results demonstrated that the VTE risk prediction model established in this study held greater clinical utility for patients with GC/EC. The Simp-GB model can provide intelligent assistance in the early clinical assessment of VTE risk in these patients.

## 1 Introduction

### 1.1 Background

Cancer, as a primary cause of mortality worldwide, has emerged as a significant impediment to the improvement of life expectancy. Existing statistical data from the World Health Organization (WHO) indicate that malignancies of the upper gastrointestinal tract constitute a substantial burden on global healthcare economies. Gastric Cancer (GC) and Esophageal Cancer (EC), as subtypes of upper gastrointestinal malignancies, exhibit certain resemblances despite differing in localization and clinical characteristics (Xie et al., 2021). GC stands as the fifth most common cancer worldwide and the fourth leading cause of cancer-related mortality globally. According to the GLOBOCAN 2020 database by the International Agency for Research on Cancer (IARC), China reported 479,000 new cases of GC in 2020, representing 45% of global incidence (Cao et al., 2021). EC ranks seventh among the most prevalent cancers globally and sixth among leading causes of death, exhibiting a notably high incidence rate in China, contributing 53.70% and 55.35% of new cases and deaths, respectively, to the global tally (Sung et al., 2021). Early symptoms of GC and EC are often indistinct, with the majority of patients diagnosed at advanced stages, typically accompanied by local or systemic metastases (Xu et al., 2020). Digestive malignancies in China, including GC, EC, and hepatic cancers, exhibit generally poor prognoses, with a 5-year overall survival rate of less than 36% based on 2018 statistics from the National Cancer Center (Allemani et al., 2018). Beyond the poor prognosis, GC and EC rank third and fourth, respectively, in terms of Disability Adjusted Life Years (DALYs) among all cancers, severely impacting societal productivity and generating significant socioeconomic burdens from a healthcare perspective (Zhou et al., 2019). According to the 2022 China Malignant Tumor Disease Burden Report, the incidence rates of GC and EC show a declining trend. However, with the rapid economic development in China, factors such as population growth, aging, increased tobacco and alcohol consumption, dietary changes, obesity, and other risk factors contribute to GC and EC remaining significant medical and public health concerns in China (Han et al., 2024).

Venous thromboembolism (VTE) represents the second leading cause of mortality in cancer patients following tumor progression, comprising pulmonary embolism (PE) and deep venous thrombosis (DVT) (Khorana et al., 2022). Apart from increasing mortality among cancer patients, VTE also leads to higher healthcare costs and complicates the treatment process (Khorana et al., 2022). Ranking third among thrombosis-related conditions following myocardial infarction and stroke, VTE has emerged as a significant public health concern, imposing a substantial disease burden (Goldhaber and Bounameaux, 2012; Gregson et al., 2019). The association between malignant tumors and hypercoagulable states was initially described by Armand Trousseau in the early 19th century. Tumor presence independently increases the risk of VTE, with cancer-afflicted adults exhibiting a 4–6.5-fold higher risk compared to the general population (Khorana et al., 2007). While the association between cancer and thrombosis formation has been established, the magnitude of risk for thromboembolic events varies across different tumor types. The incidence of VTE among populations with GC and EC ranges from 9% to 20%, with GC patients demonstrating a higher incidence compared to EC patients (17.8% vs. 13.4%) (Pfrepper, 2020; Marshall-Webb et al., 2016).

### 1.2 Influence factors

#### 1.2.1 Patient factors

The risk of VTE in cancer patients is associated with patient-specific characteristics. Current literature indicates that males have a higher risk of VTE compared to females, although environmental and acquired factors may be the primary contributors to the difference between the two genders (Ciarambino et al., 2023). Age is also a contributing factor to the increased risk of DVT, partly due to the increased prevalence of other risk factors among the elderly population. Additionally, smoking and obesity are both associated with a higher risk of VTE (Cohen and Bistervels, 2021). Several chronic diseases are also associated with the occurrence of VTE, including atherosclerosis, congestive heart failure, hypertension, dyslipidemia, chronic kidney disease, rheumatoid arthritis, severe infections such as sepsis, asthma, and diabetes. In addition to patient demographics and disease status, VTE also carries a genetic risk, including deficiencies in anticoagulant enzymes, protein C or protein S gene (Hu et al., 2022).

#### 1.2.2 Tumor factors

Tumors represent a significant risk factor for thrombosis, with the mechanisms underlying thrombus formation being complex and involving the interaction of various factors, such as the generation of plasminogen activator inhibitor-1 (PAI-1), the release of tissue factor (TF), and the production of cytokines by tumor cells (Hiraide et al., 2020). For GC or EC patients, the staging and histological classification of tumors also influence the magnitude of VTE risk. Statistics reveal that patients with stage IV tumors have a higher risk of VTE, with a relative risk (RR) of 1.9 and a 95% confidence interval (CI) of 1.6–2.3 (Blom et al., 2006). Furthermore, distant metastasis of tumor cells similarly increases the risk of VTE. Blom et al. reported that the adjusted odds ratio (OR) for VTE risk in patients with solid malignant tumors with distant metastases compared to those without advanced metastasis was 19.8 (Blom et al., 2005). Additionally, research suggests that adenocarcinoma has a closer association with VTE compared to other pathological types (Noble and Pasi, 2010).

#### 1.2.3 Treatment factors

Factors related to treatment strategies influence thrombosis risk in cancer patients. Firstly, prolonged bed rest during hospitalization leads to local stasis due to the loss of muscle pump function, activating the coagulation system and promoting thrombus formation (Hu et al., 2022). Secondly, it has been reported that the incidence of VTE in patients with GC and hepatobiliary pancreatic tumors can reach 25% if anticoagulation is not prophylactically administered post-surgery (Larsen et al., 2015). The occurrence of VTE after esophagectomy ranges from 5% to 7%, with a twofold increase in the risk of mortality (Zwischenberger et al., 2016). For malignant tumor patients requiring chemotherapy, the use of central venous access devices (CVADs) can enhance their quality of life and satisfaction by preventing damage from repeated venipunctures and from irritating medications, while may also damage the vascular endothelium, leading to thrombus formation (Akhtar and Lee, 2021; Achinger and Ayus, 2019). Furthermore, radiotherapy is an important treatment modality for cancer patients, although current research focuses more on the relationship between radiotherapy and damage to arterial endothelial cells, with fewer investigations into the risk of VTE associated with radiotherapy. A study involving 450 radiotherapy-treated cancer patients found a cumulative incidence of VTE at 6 months of approximately 2% (95% CI: 0.9–3.7), with no significant association between radiotherapy and VTE risk observed. Therefore, further research is needed to elucidate the relationship between radiotherapy and VTE(Daguenet et al., 2022).

Platinum-based therapies are the most frequently associated with increased VTE risk among the chemotherapy drugs. A meta-analysis of a randomized controlled trial (RCT) involving 8,216 patients with advanced solid tumors demonstrated a 1.7-fold increase in VTE risk in patients receiving cisplatin chemotherapy (Seng et al., 2012). Studies have reported a significantly increased risk of VTE associated with cisplatin compared to oxaliplatin when used in combination chemotherapy for GC or EC (7.6% vs. 15.1%, P < 0.001) (Cunningham et al., 2008). It has recently been demonstrated that platinum compounds and/or gemcitabine are significantly associated with increased VTE risk (Roselli et al., 2013). Furthermore, fluoropyrimidine-based treatment regimens may also induce acquired thrombotic disorders, leading to VTE during chemotherapy. Research has shown that the incidence of VTE in colorectal cancer patients treated with 5-fluorouracil (5-FU) and granulocyte colony-stimulating factor (G-CSF) reaches 29% (Tournigand et al., 2004).

Apart from anticancer medication, supportive therapies may also increase the risk of thrombosis, such as transfusions and erythropoiesis-stimulating agents. Additionally, the risk of VTE formation and recurrence induced by vascular endothelial growth factor receptor (VEGFR) inhibitors increases by 6-fold and 2-fold, respectively, with a maximum incidence rate of 11% (Mihalcea et al., 2023). Lastly, a substantial amount of data has demonstrated an association between immune checkpoint inhibitors (ICIs) and thromboembolic events, although data indicating potential causality are currently lacking.

### 1.3 The current status of predictive models

Cancer Associated Thrombosis (CAT) is a multifactorial disease, and for GC and EC patients, effective identification and management of high-risk VTE patients are crucial for preventing thrombotic events and improving life quality. With the development of big data technology and the improvement of hospital health information systems, artificial intelligence (AI) is gradually being applied in clinical management. Machine learning is one of the core technologies of AI, which builds predictive models through a large amount of data. Predictive models in healthcare systems can utilize statistical tools based on individual patient data (such as demographics, clinical history and tests) to assess the possibility of events such as VTE occurring within a specific timeframe (Steyerberg et al., 2013).

The Risk Assessment Model (RAM) is a clinical decision-making tool. Risk prediction models based on RAM aid clinicians in making appropriate anticoagulation decisions for VTE patients. RAM, driven by clinical needs, serves as an extension and complement to RCTs, providing essential scientific evidence for clinical decision-making through research analysis of a broader patient population. Over the past decade, several models (or scales) have been developed to guide physicians in assessing VTE risk in patients. Among them, the Caprini (Caprini, 2005) and Padua (Barbar et al., 2010) scoring models have been widely validated and utilized both domestically and internationally. Additionally, the Khorana score (Khorana et al., 2008) and COMPASS-CAT(Gerotziafas et al., 2017) are risk assessment tools developed for VTE risk in cancer patients across various clinical settings. However, the applicability of these assessment scales for gastric and esophageal cancer patients has not been adequately demonstrated.

GC or EC patients face heightened risks of VTE and bleeding, rendering clinical anticoagulation decisions more intricate and necessitating personalized treatment strategies tailored to individual circumstances. This study collected medical and health information of GC/EC patients to analyze and evaluate VTE events occurring within the initial 6 months post-hospital admission. A retrospective assessment was conducted on factors influencing VTE occurrence in GC/EC patients, and leveraging machine learning algorithms, an interpretable risk prediction model was constructed to forecast the risk of VTE among these patients. Employing the optimal model derived from this study, a comparative analysis was conducted against several benchmark evaluation metrics to explore the predictive performance advantages of the proposed model. Additionally, exploratory validation was performed utilizing the study data, and external validation was conducted to assess their predictive efficacy specifically within the context of GC/EC patients in China.

## 2 Materials and methods

### 2.1 Data explanation

The data for this study were obtained through the electronic health record system (EHRS) of Sichuan Provincial People’s Hospital and telephone follow-up. All participants were inpatients, with inclusion criteria as follows: (1) age ≥18 years; (2) histologically confirmed diagnosis of GC or EC. The diagnostic criteria adhered to the definitions by the Chinese Society of Clinical Oncology (CSCO) for GC (2022 edition) and EC (2020 edition). The following exclusion criteria were applied: (1) GC/EC not being the primary tumor or presence of primary malignant tumors at multiple sites; (2) prior anti-cancer treatment received at other medical institutions after tumor diagnosis; (3) occurrence of VTE before the diagnosis of the malignancy; (4) hospital stay of less than 48 h or incomplete hospitalization records; (5) difficulty in obtaining VTE event records within 6 months post-admission due to loss to follow-up or refusal of follow-up.

During the research process, the personal information of patients, such as names, home addresses, and contact approaches, was anonymized. This study has received approval from the ethics committee of Sichuan Provincial People’s Hospital, with the review certification provided in Supplementary Appendix A.

### 2.2 Variable description

76 input variables were identified based on literature review and analysis of actual medical records, including demographic information, medical history, laboratory indicators, tumor-related characteristics, and information on pharmacological or non-pharmacological treatments. The outcome variable of this study is the occurrence of VTE within 6 months of hospitalization in patients with GC/EC.

### 2.3 Variable screening

Variables with more than 90% missing values, a maximum percentage of records in a single category exceeding 90%, or a maximum number of categories surpassing 90% were excluded. The minimum coefficient of variation was set at 0.1, and the minimum standard deviation was set at 0.

### 2.4 Data partitioning

After variable selection, the dataset was randomly divided into a training set and a testing set in an 8:2 ratio, ensuring that the proportions of patients with different labels remained consistent between the training and testing sets. The model was constructed using the training set, while the testing set was used for performance evaluation after the modeling phase.

### 2.5 Model algorithms

This study utilized a total of nine supervised learning algorithms for modeling, including Logistic Regression (LR), Support Vector Machine (SVM), K-Nearest Neighbors (KNN), Random Forest (RF), Extreme Gradient Boosting (XGBoost), Adaptive Boosting (AdaBoost), Light Gradient Boosting Machine (LightGBM), Categorical Boost (CatBoost), and Gradient Boosting (GBoost).

To accurately track and document the performance, parameters, training data, and other information regarding the models, this study assigned unique identifiers (IDs) to the data imputation methods, data sampling techniques, feature selection approaches, and machine learning algorithms employed. The data cleaning methods and summaries of these algorithms are presented in Table 1.

**TABLE 1:** Summary of data cleaning methods and algorithms in machine learning.

Operations	Methods	Parameters	ID
Data Imputation	simple imputation	Simple	0
	KNN imputation	KNN	1
	ISVD imputation	ISVD	2
	RF imputation	RF	3
Data Sampling	ROS	ROS	0
	SMOTE	SMO	1
	SMOTEb	BSMO	2
	SMOTEN	SMN	3
Feature Selection	Lasso regression	LA	0
	Ridge regression	RD	1
	Boruta regression	BOR	2
	ElasticNet regression	EN	3
Algorithms	LR	LR	0
	SVM	SVM	1
	KNN	KNN	2
	RF	RF	3
	XGBoost	XGB	4
	AdaBoost	AB	5
	LightGBM	LB	6
	CatBoost	CB	7
	GBoost	GB	8

AdaBoost, Adaptive Boosting; GBoost, Gradient Boosting; ISVD, iterative singular value decomposition; LightGBM, light gradient boosting machine; LR, logistic regression; RF, random forest; ROS, random over sampling; SMOTE, synthetic minority oversampling technique; SMOTEb, borderline-SMOTE; SMOTEN, SMOTE, for Nominal; SVM, support vector machine; XGBoost, Extreme Gradient Boosting.

### 2.6 Model evaluation

In assessing the predictive performance of the models, the following performance metrics were employed: accuracy, precision, recall, F1 score, area under the ROC curve (AUC), and area under the precision-recall curve (PRAUC).

### 2.7 Variable importance

The variable importance reflects the contribution of input variables to the outcome variable in a specific model. In this study, SHAP is employed for quantification. Within the SHAP plot, each row represents a specific feature, while the horizontal axis corresponds to the SHAP values of that feature.

## 3 Results

### 3.1 Research population

**FIGURE 1:**
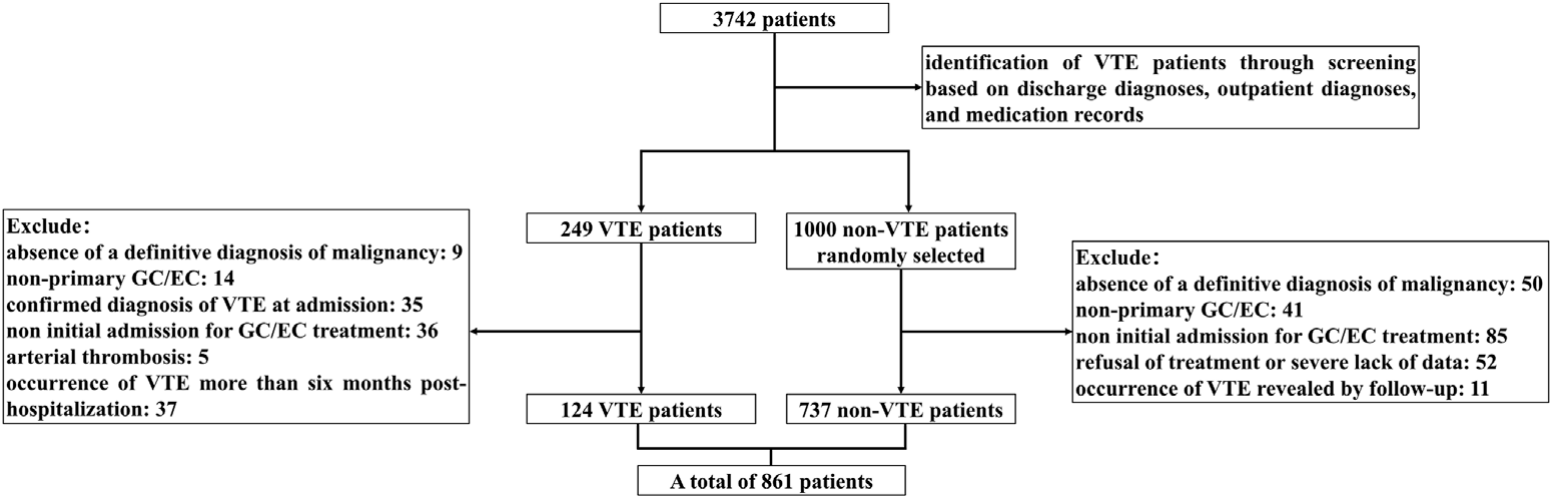
Patient inclusion flow chart.

A total of 7,539 medical records of hospitalized patients diagnosed with GC/EC were extracted and 3,742 cases remained after excluding duplicate ones. Among these, 249 patients with confirmed VTE were identified from both inpatient and outpatient diagnostic records. Subsequently, 1,000 patients without diagnosed VTE were randomly selected from the remaining population. Following the inclusion and exclusion criteria, which involved the exclusion of minors, cases with unclear tumor diagnosis, non-initial hospitalizations for treatment, refusal of treatment or missing data, patients already diagnosed with VTE upon admission, loss to follow-up, or unwillingness to cooperate with follow-up, a final cohort comprising 861 patients was established. Among these, 124 cases experienced VTE, while 737 cases did not. The process of case selection is illustrated in Figure 1.

### 3.2 The results of variable screening

Medical record information of enrolled patients was collected and analyzed. All variables were extracted from objective clinical data from the hospital information system. Among them, lower limb edema is assessed by clinicians and nurses, and the evaluation criteria for preventive anticoagulation in cancer patients is that the patient has used anticoagulant drugs during treatment without a confirmed diagnosis of thrombosis. After data cleansing, a total of 76 feature variables were obtained, including 47 qualitative variables and 29 quantitative variables. Descriptive analyses were performed on the data, with statistical results for the qualitative variables presented in Table 2 and results for the quantitative variables shown in Table 3.

**TABLE 2:** Descriptive statistical analysis of qualitative variables.

Variables	Categories	Num	%	Variables	Categories	Num	%
Gender				Histological grade of tumor			
	Male	600	69.7		Phase Ⅰ	125	14.5
	Female	261	30.3		Phase Ⅱ	188	21.8
Ethnic					Phase Ⅲ	231	26.8
	Han	769	89.3		Phase Ⅳ	178	20.7
	Minority	92	10.7		Unknown	139	16.2
Family history of malignancy				Tumor invasion			
	Yes	74	8.6		Yes	583	8.4
	No	787	91.4		No	72	67.7
History of smoking					Unknown	206	23.9
	Yes	313	36.4	Degree of tumor differentiation			
	No	548	63.6		Low	239	27.8
History of alcohol consumption					Middle	291	33.8
	Yes	263	30.5		High	23	2.6
	No	598	69.5		Unknown	308	35.8
History of blood transfusion				Lymph node metastasis			
	Yes	118	13.6		Yes	534	62.0
	No	743	86.3		No	246	28.6
Diabetes					Unknown	81	9.4
	Yes	88	10.2	Distant metastases			
	No	773	89.8		Yes	181	21.0
Hypertension					No	597	69.3
	Yes	200	23.3		Unknown	83	9.7
	No	661	76.7	Use of platinum-based drugs			
Other cardiovascular diseases					Yes	142	16.5
	Yes	96	11.1		No	719	83.5
	No	765	88.9	Use of Fluorouracils			
Lung disease					Yes	146	17.0
	Yes	59	6.9		No	715	83.0
	No	802	93.1	Use of VEGFR inhibitors			
Alzheimer’s disease					Yes	38	4.4
	Yes	3	0.3		No	823	95.6
	No	858	99.7	Use of Capecitabine			
Edema of the lower extremities					Yes	69	8.0
	Yes	14	1.6		No	792	92.0
	No	847	98.4	Glucocorticoid replacement therapy			
Ascites					Yes	43	5.0
	Yes	142	16.5		No	818	95.0
	No	719	83.5	Use of Antiplatelet drugs			
Varicose veins of the lower extremities					Yes	63	7.3
	Yes	14	1.6		No	798	92.7
	No	847	98.4	Use of Erythropoietin/GGF			
Severe infection					Yes	103	12.0
	Yes	73	8.5		No	758	88.0
	No	788	91.5	Prophylactic anticoagulation			
Ileus					Yes	195	22.6
	Yes	35	4.1		No	666	77.4
	No	826	95.9	Use of hemostatic drugs			
Hyperlipidemia					Yes	95	11.0
	Yes	27	3.1		No	766	89.0
	No	834	96.9	Underwent tumor eradication surgery			
Autoimmune diseases					Yes	611	71.0
	Yes	12	1.4		No	250	29.0
	No	849	98.6	Type of surgery			
History of VTE disease					Open surgery	404	66.2
	Yes	3	0.3		Laparoscopic surgery	207	33.8
	No	858	99.7	Blood transfusions during surgery			
History of surgery					Yes	95	11.0
	Yes	25	2.9		No	766	89.0
	No	836	97.1	Neoadjuvant chemotherapy			
Hepatic insufficiency					Yes	23	97.3
	Yes	29	3.4		No	838	2.7
	No	832	96.6	Radiation therapy			
Renal insufficiency					Yes	105	12.2
	Yes	25	2.9		No	756	87.8
	No	836	97.1	Intravenous catheter devices			
History of glucocorticoid use					Yes	278	14.4
	Yes	0	0		No	583	85.6
	No	861	100	Type of tumor			
Tumor histological type					GC	720	83.6
	Adenocarcinoma	590	68.5		EC	130	15.1
	Squamous cell carcinoma	144	16.7		Others	11	1.3
	Gastrointestinal stromal tumors	31	3.6				
	Others	96	11.2				

GGF, granulocyte growth factor; VEGFR, vascular endothelial growth factor receptor; VTE, Vous thromboembolism.

**TABLE 3:** Descriptive statistical analysis of quantitative variables.

Variables	Num	Min	Max	Mean	SD
Age	858	27.00	91.00	63.86	10.955
BMI	832	12.94	32.88	22.32	3.241
Bathel score	850	5.00	100.00	92.33	14.920
white blood cell	828	1.25	39.86	6.27	3.137
neutrophilicgranulocyte	829	0.83	88.00	4.35	4.114
monocyte	827	0.01	123.00	0.61	4.280
red blood cell	829	0.49	123.00	4.134	4.20
platelet	830	4.48	799.00	212.61	91.151
hemoglobin	830	0.36	197.00	115.19	28.379
hematocrit	831	0.14	262.00	34.84	13.049
albumin	828	20.70	79.70	34.44	5.280
creatinine	829	4.50	984.90	72.20	41.548
INR	810	0.81	80.40	1.19	2.991
APTT	813	0.95	71.30	26.95	3.562
APTR	699	0.73	27.40	1.04	1.151
PT	815	0.89	30.60	11.43	1.481
TT	814	0.19	43.20	17.02	1.712
Fibrinogen	815	0.89	16.40	3.45	1.177
FDP	637	0.30	282.70	5.09	13.208
D-dimer	647	0.02	92.87	1.93	5.813
hs-CRP	680	0.11	262.51	16.81	34.899
hs-TnI	314	0.00	14,386.60	60.24	817.989
CEA	769	0.37	15,000.00	65.92	756.078
CA 19–9	757	0.80	72,000.00	467.61	3,102.055
CA 125	725	3.00	4,501.90	54.31	248.871
CA 72–4	463	0.34	500.00	21.67	58.401
CA 242	66	0.58	97.42	7.33	15.864
HDL-C	389	0.34	2.30	1.15	0.325
Maximum tumor diameter	582	0.20	15.00	4.56	2.581
Date of surgery	481	1.00	12.00	4.74	1.678

APTR, activated partial thromboplastin ratio; APTT, activated partial thromboplastin time; BMI, body mass index; CA, carbohydrate antigens; CEA, carcinoembryonic antigen; FDP, fibrinogen degradation products; hs-CRP, hypersensitive-C reactive protein; hs-TnI, high-sensitivity troponin I; INR, international normalized ratio; PT, prothrombin time; TT, thrombin time.

Out of the 76 input variables, 20 were excluded due to data imbalance (one had a high proportion of missing values, and 19 had high proportions in single categories). The remaining 56 variables were used for modeling, referred to as full-variable modeling. A simplified modeling approach was then applied using the top 12 variables ranked by feature importance from the best model obtained through full-variable modeling.

### 3.3 Model building and evaluation

#### 3.3.1 Full-variable modeling

Through the combination of four data imputation methods, four data sampling methods, and four feature selection methods, a total of 64 datasets were generated. Nine machine learning algorithms were applied to model these 64 datasets, resulting in 576 machine learning models. Model training was conducted while adjusting internal parameters to optimize the performance of each model. The performance metrics of all tuned models are presented in Supplementary Appendix B.

**FIGURE 2:**
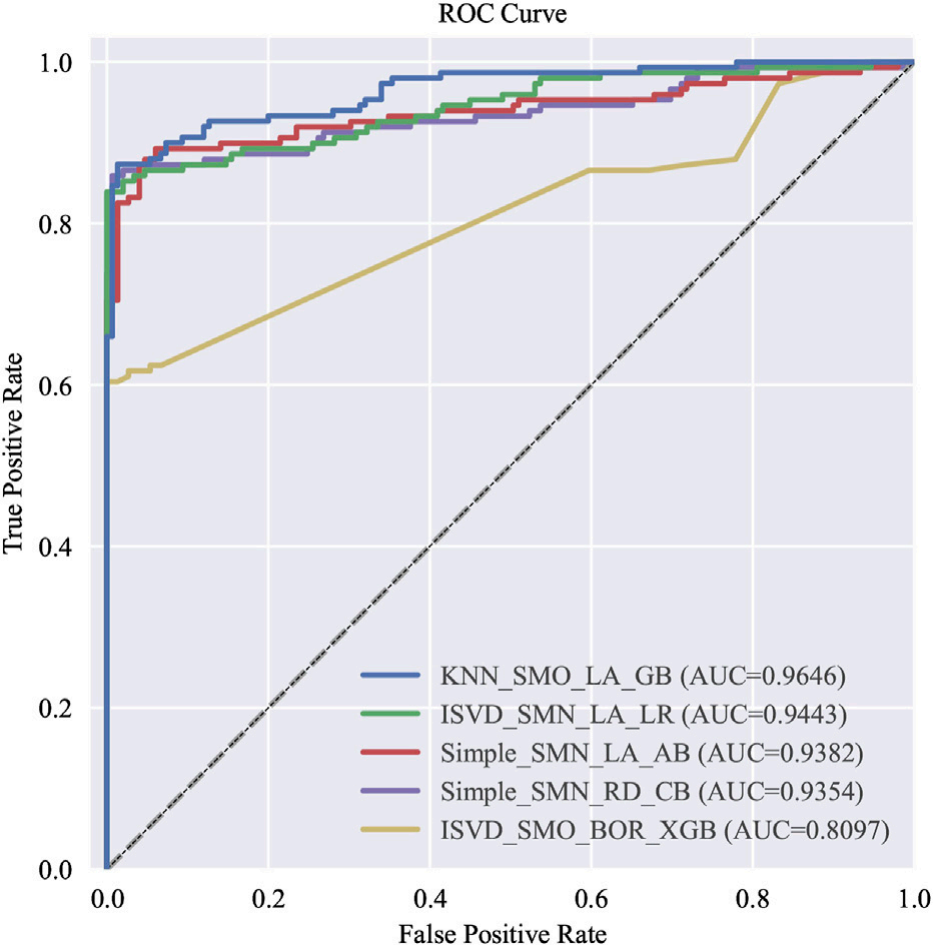
The ROC curve of T5Ms-F.

**FIGURE 3:**
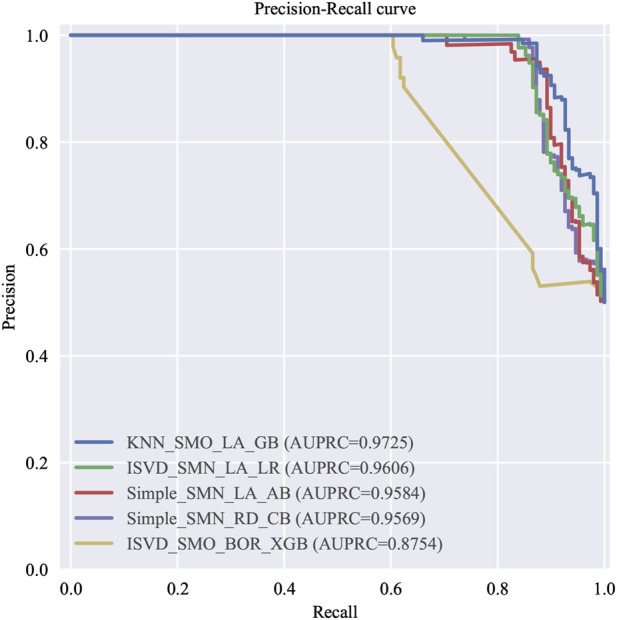
The P-R curve of T5Ms-F.

Among the 576 predictive models established based on the full variables, five optimal models were selected according to various evaluation criteria. These models are identified as follows: the one with the highest AUC, the one with the highest AUC in logistic regression, the one with the highest recall rate, the one with the highest F1 score, and the one with the fewest included features, which are hereinafter referred to as the top five models with full variables (T5Ms-F). The ROC curves and P-R curves of T5Ms-F are respectively illustrated in Figures 2, 3. The summary of predictive performance for T5Ms-F is provided in Table 4. Through a combined analysis of the ROC curve and PR curve for the model, it was determined that the model established based on the KNN imputation method, SMOTE oversampling technique, and LASSO regression feature selection method, and subsequently employing the Gradient Boosting algorithm, emerged as the optimal model for comprehensive variable modeling, denoted as GB Model. It exhibits an AUC of 0.9646 and a recall rate of 0.8267.

**TABLE 4:** The evaluation results of T5Ms-F.

Data imputation	Data sampling	Feature selection	Algorithm	AUC	Accuracy	Precision	Recall	F1 score
1	1	0	8	0.9646	0.9100	0.9920	0.8267	0.9018
2	3	0	0	0.9443	0.8993	0.9281	0.8658	0.8958
0	3	0	5	0.9382	0.9060	0.9172	0.8926	0.9048
0	3	1	7	0.9354	0.9228	0.9773	0.8658	0.9181
2	1	2	4	0.8097	0.7785	0.9029	0.6242	0.7381

Specific methods for data imputation, data sampling, feature selection and algorithm were displayed with ID, values (Table 1).

T5Ms-F, the top five models with full variables.

#### 3.3.2 Simplified modeling

**FIGURE 4:**
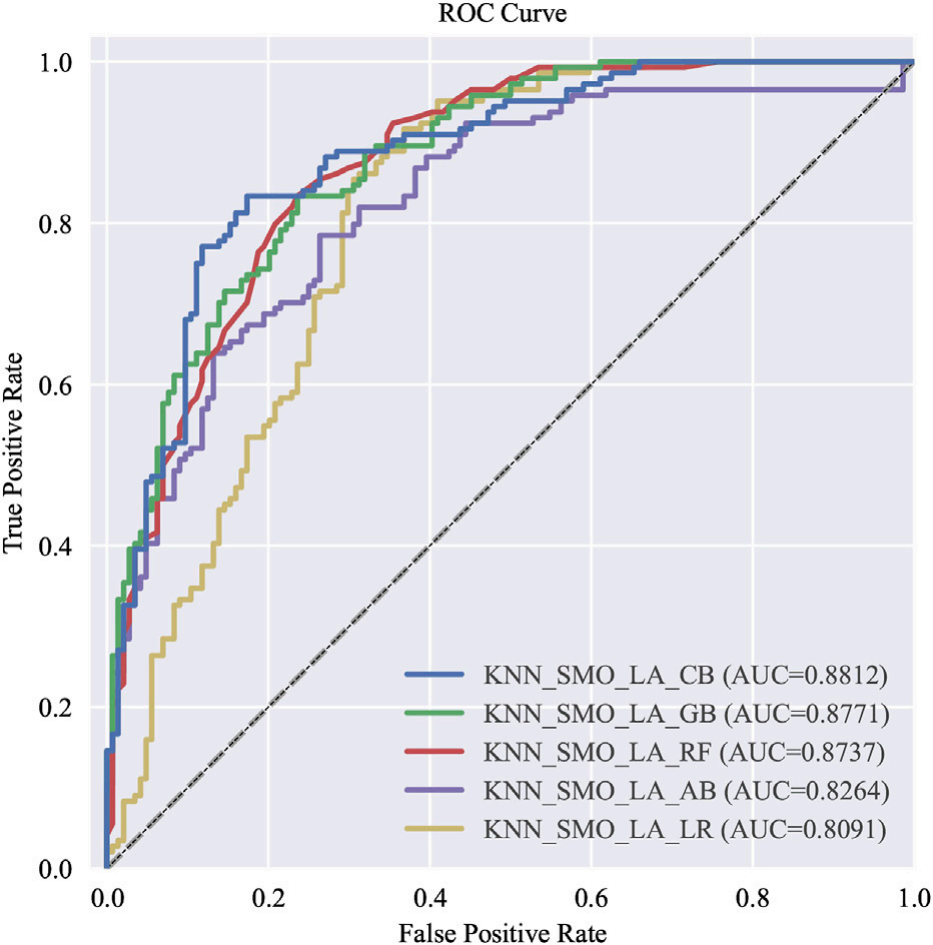
The ROC curve of T5Ms-S.

**FIGURE 5:**
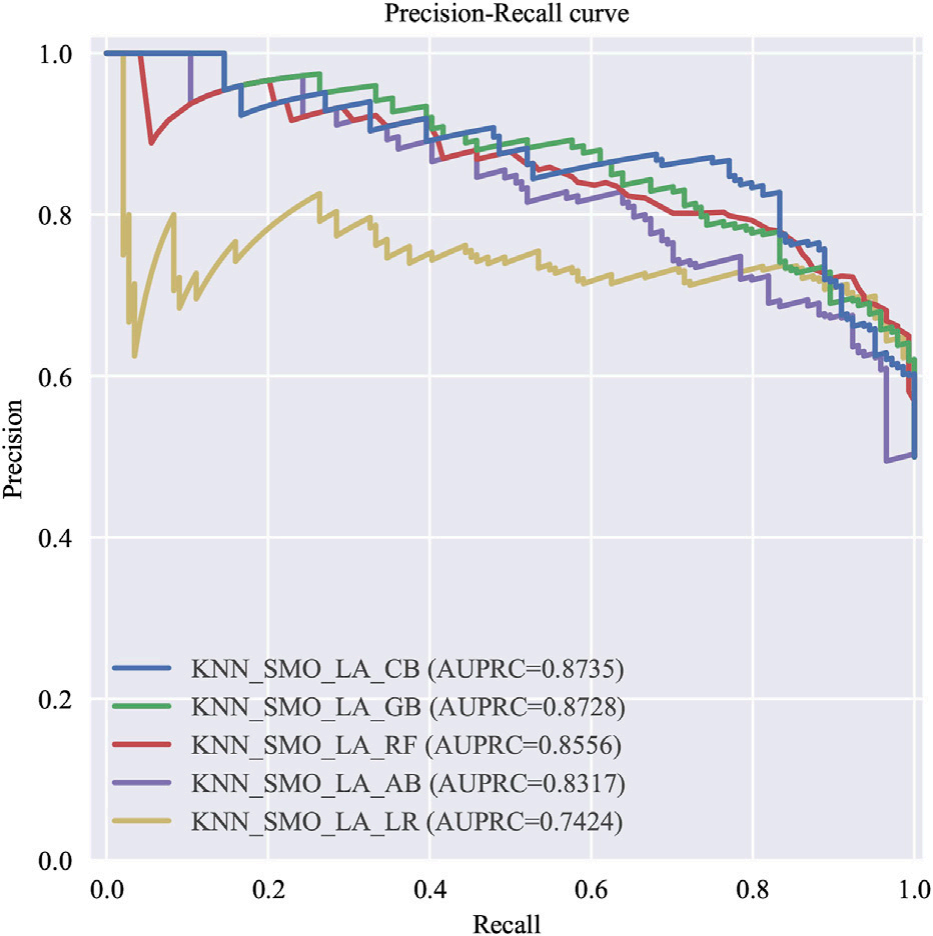
The P-R curve of T5Ms-S.

Simplified models were built based on the top 12 variables ranked by feature importance in the Gradient Boosting model with nine machine learning algorithms. Following, parameter optimization was conducted. The optimal five predictive models were sequentially identified as follows: the one with the highest AUC value, the one with the highest F1 score, the one with the highest precision, the one with the highest recall rate, and the one with the highest AUC value established through logistic regression modeling, which abbreviated hereafter as the top five models with simplified variables (T5Ms-S). ROC and P-R curves were plotted for each of T5Ms-S. The evaluation metrics for T5Ms-S, ranked by performance, are presented in Table 5 while the ROC and P-R curves are depicted in Figures 4, 5, respectively.

**TABLE 5:** The evaluation results of T5Ms-S.

Models	Variables	AUC	Accuracy	Precision	Recall	F1 score
Simp-CatBoost	12	0.8811	0.7430	0.8571	0.5833	0.6942
Simp-GradientBoost	12	0.8771	0.7604	0.8504	0.6319	0.7251
Simp-RandomForest	12	0.8736	0.7222	0.8556	0.5347	0.6581
Simp-AdaBoost	12	0.8263	0.7430	0.7822	0.6736	0.7239
Simp-Logistic Regression	12	0.8090	0.6944	0.7258	0.6250	0.6716

T5Ms-S, the top five models with simplified variables.

In this study, recall rate specifically refers to the proportion of patients who developed VTE within 6 months and were successfully identified. The improvement in recall implies enhanced predictive capability for high-risk VTE in patients with GC/EC, thereby aiding in reducing cases of high-risk patients being missed. In the simplified models, although the Simp-CatBoost model exhibited the highest AUC performance, with a value of 0.881, its recall rate was relatively low at 0.5833. Conversely, while the AUC of the Simp-Gradient Boost model was slightly lower than that of the Simp-CatBoost model (0.877 vs. 0.881), its recall rate and F1 score were higher (recall = 0.6319, F1 score = 0.7251). Therefore, considering all evaluation metrics, the Simp-Gradient Boost model (hereinafter referred to as the Simp-GB Model) emerged as the optimal simplified model.

#### 3.3.3 Model performance comparison

**FIGURE 6:**
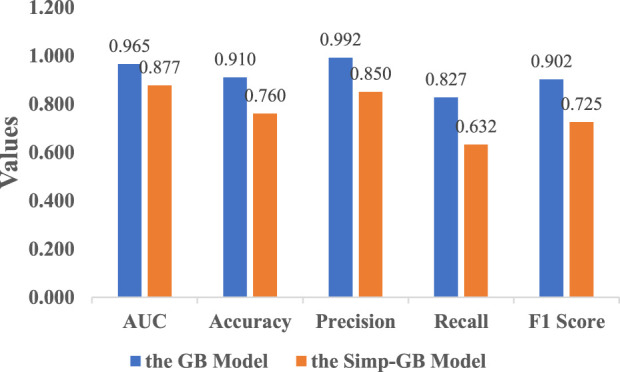
A comparison between the optimal models of full-variable modeling and simplified modeling.

Comparison between the optimal models of full-variable modeling and simplified modeling, namely, the GB Model and the Simp-GB Model, is conducted. The results demonstrate that the predictive performance of the Simp-GB Model is inferior to that of the full-variable modeling model. The comparative evaluation metrics of the two models are illustrated in Figure 6. Although the predictive performance of the GB model surpasses that of the Simp-GB model, the Simp-GB model, constructed based on 12 feature variables, is selected as the optimal risk prediction model for this study due to its clinical practicality and generalizability.

### 3.4 Analysis of feature importance

**FIGURE 7:**
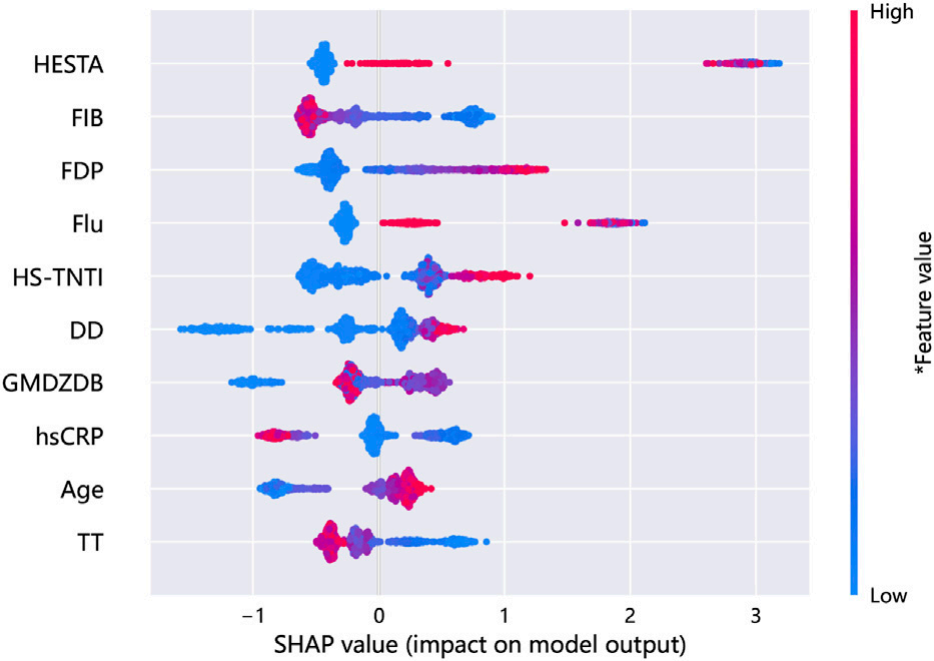
A comprehensive SHAP summary plot for the full-variable modeling.

The SHAP diagram illustrating the incorporation of full variables in the establishment of the risk prediction model for VTE in patients with GC/EC is presented in Figure 7. The results indicate that among the included variables, hemostatic drugs, fibrinogen, FDP, fluoropyrimidine drugs, hs-TNTI, D-D, HDL, hs-CRP, age, and TT are the top 10 variables contributing significantly to the model. According to the findings depicted in Figure 7, FDP, fluoropyrimidine drugs, hs-TNTI, D-D, and age exhibit a positive correlation with the predicted incidence of VTE, whereas FIB, hs-CRP, and TT demonstrate a negative correlation with VTE incidence. The influence trend of HDL on the prediction outcome is less evident. In summary, hemostatic drugs exert the greatest impact on the occurrence rate of VTE within GB Model.

**FIGURE 8:**
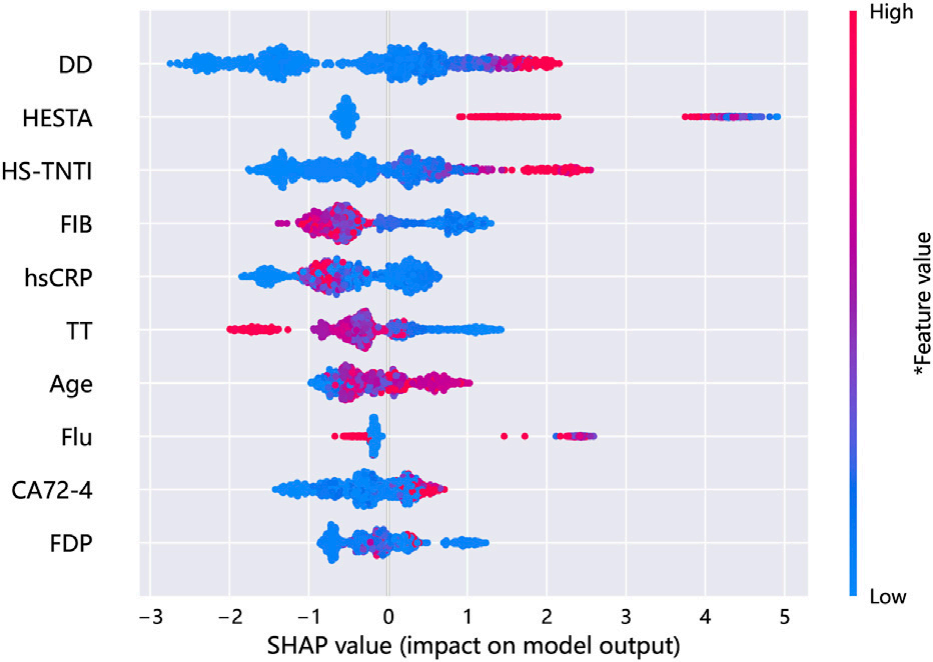
A comprehensive SHAP summary plot for the simplified-variable modeling.

The SHAP diagram illustrating the incorporation of selected variables in the establishment of the risk prediction model for VTE in patients with GC/EC is presented in Figure 8. The results indicate that D-D hemostatic drugs, hs-TNTI, FIB, hs-CRP, TT, age, fluoropyrimidine drugs, carbohydrate antigen 72–4, and FDP are the top 10 variables contributing most significantly to the Simp-GB model. As illustrated in Figure 8, D-D, hemostatic drugs, hs-TNTI, age, fluoropyrimidine drugs, and carbohydrate antigen 72–4 exhibit positive correlations with the predicted incidence of VTE, while FIB, hs-CRP, and TT show negative correlations. The influence trend of FDP on the prediction is inconspicuous. In summary, D-D has the most significant impact on the incidence of VTE in Simp-GB Model.

### 3.5 External validation of four risk assessment scales

Following multidimensional comparisons, the Simp-GB Model was selected as the final model for this study, exhibiting an AUC value of 0.877, indicating good predictive performance. Utilizing the test set employed in constructing Simp-GB Model, external validation was conducted on four previously validated predictive models, namely, Caprini, Padua, Khorana, and COMPASS-CAT. Scores were computed according to the respective model specifications, and samples were stratified based on these scores to assess risk levels. Given that test set of the Simp-GB Model comprised only 12 variables, rendering accurate score calculations unfeasible, external validation was performed using the test set of the GB Model, consisting of 300 instances, comprising 150 positive and 150 negative samples, encompassing 46 features.

#### 3.5.1 Risk stratification results

According to the Khorana score, 20% of VTE occurrences were observed in the low-risk group (n = 30), 70.7% in the moderate-risk group (n = 106), and only 9.3% of VTE incidents were attributed to the high-risk group (n = 14). The proportion differences of VTE occurrences across different risk strata were not statistically significant (P = 0.077), as illustrated in Table 6 depicting the risk stratification outcomes.

**TABLE 6:** Stratified outcomes of VTE across various scoring scales.

Scales	All patients (%)	VTE patients (%)	χ^2^ value	P value
Khorana	300	150		
Low-risk (0)	46 (15.3)	30 (20)	5.128	0.077
Medium-risk (1–2)	226 (75.4)	106 (70.7)		
High-risk (≥3)	28 (9.3)	14 (9.3)		
Caprini	300	150		
Medium-risk (2)	3 (1.0)	3 (2.0)	79.747	<0.001
High-risk (3–4)	60 (20.0)	60 (40.0)		
Extreme-risk (≥5)	237 (79.0)	87 (58.0)		
Padua	300	150		
Low-risk (0–3)	211 (70.3)	115 (76.7)	5.767	0.016
High-risk (≥4)	89 (29.7)	35 (23.3)		
COMPASS-CAT	300	150		
Low-risk (0–6)	241 (80.3)	141 (94.0)	35.467	<0.001
High-risk (≥7)	59 (19.7)	9 (6.0)		

Due to the presence of cancer as one of the risk factors in the Caprini score, none of the patients in this study were defined as low VTE risk (i.e., Caprini score 0–1). Among the population experiencing VTE, 2.0% occurred in the moderate-risk group (n = 3), 40% in the high-risk group (n = 60), and 58% in the extremely high-risk group (n = 87). According to the chi-square test, the overall distribution disparity of VTE events was statistically significant (P < 0.05). Due to all three patients in the moderate-risk group experiencing VTE, a chi-square test for this group could not be conducted. Ultimately, a comparison was made between the distribution disparities of the high-risk and extremely high-risk groups, revealing a higher proportion of VTE occurrences in the extremely high-risk group compared to the high-risk group, with statistically significant differences (58% vs. 40%, P < 0.001). This suggests a positive correlation between Caprini scores and the risk of VTE incidence in patients with GC/EC.

According to the Padua score, 76.6% of the VTE-positive patients were classified as low risk (n = 115), while only 23.3% of which were classified as high risk (n = 35). Based on the results of the chi-square test, the proportion of VTE occurrences in the low-risk group was significantly higher than that in the high-risk group, with a statistically significant difference (76.6% vs. 23.3%, P < 0.05). This indicates that if the Padua score is used for VTE risk stratification, a large number of VTE high-risk individuals will be missed.

The COMPASS-CAT model exhibits similarities with the Padua score, with significantly more patients in the low-risk group compared to the high-risk group (94% vs. 6%, P < 0.05). This implies that in the COMPASS-CAT model, 94% of GC/EC patients were unable to receive adequate prevention, suggesting that for the VTE group, the accuracy of risk stratification by the COMPASS-CAT score is diminished, rendering it incapable of properly distinguishing between low-risk and high-risk patients.

#### 3.5.2 Comparison across various models

To preliminarily analyze the score disparities between the VTE and non-VTE groups across the four scales, independent t-tests were conducted for statistical comparison. Results indicated that, in the Caprini score, the mean score for the VTE group (5.82 ± 2.704) was lower than that of the non-VTE group (9.17 ± 1.421), with a statistically significant difference (P < 0.05). Similarly, in the Khorana score, the mean score for the VTE group (1.68 ± 0.898) was lower than that of the non-VTE group (1.88 ± 0.746), with a statistically significant difference (P < 0.05). Likewise, the mean score of the COMPASS-CAT assessment in the VTE group was lower than that of the non-VTE group (5.82 vs. 9.17, P < 0.05). Among these four models, only the Padua model demonstrated a significantly higher mean score in the VTE group (3.78 ± 0.874) compared to the non-VTE group (3.45 ± 0.586) (P < 0.05), as detailed in Table 7.

**TABLE 7:** Comparative analysis of scales between the VTE group and Non-VTE group.

Scales	Cohorts	N	Mean	Sd	T Value	P Value
Khorana	VTE	150	1.68	0.898	2.097	<0.001
	non-VTE	150	1.88	0.746		
COMPASS-CAT	VTE	150	5.62	2.313	7.914	<0.001
	non-VTE	150	8.37	3.567		
Caprini	VTE	150	5.82	2.704	13.415	<0.001
	non-VTE	150	9.17	1.421		
Padua	VTE	150	3.78	0.874	−3.803	<0.001
	non-VTE	150	3.45	0.586		

#### 3.5.3 Cross-sectional comparison

Cut-off values were determined based on the details in each scoring scale, and the risk classification was converted into binary values using a positive threshold. The number of true positives (TP), false positives (FP), true negatives (TN), and false negatives (FN) for each model were recorded, and accuracy, precision, recall, and F1 score were calculated based on the results. These metrics were then compared with the GB Model and the Simp-GB Model established in this study.

For the Caprini score, due to only three individuals being defined as moderate-risk in the validation dataset of this study, the moderate-risk and high-risk groups were merged, and a Caprini score of 5 was determined as the positive threshold. Regarding the Khorana score, both the American Society of Clinical Oncology (ASCO) guidelines (Key et al., 2020) and the National Comprehensive Cancer Network (NCCN) guidelines for cancer-associated VTE disease (Key et al., 2020) suggest defining patients with a Khorana score of ≥2 as high-risk. A recent systematic analysis included 27,849 cancer patients and calculated the incidence of VTE within 6 months of initial cancer diagnosis to validate the risk stratification performance of a Khorana score threshold of 3 points. The study results showed that the incidence of VTE in the low-risk group (Khorana score = 0), moderate-risk group (Khorana score = 1–2), and high-risk group (Khorana score ≥3) was 5.0% (95% CI: 3.9–6.5), 6.6% (95% CI: 5.6–7.7), and 11.0% (95% CI: 8.8–13.8), respectively. Compared to the low/moderate-risk groups, patients in the high-risk group had a relative risk of VTE within 6 months of 1.8 (95% CI: 1.5–2.1), with statistically significant differences, indicating that a Khorana score of ≥3 may have clinical significance. This study comprehensively analyzed the Khorana score by calculating predictive performance metrics using thresholds of 2 and 3 points, respectively. The results of various model performance metrics are presented in Table 8.

**TABLE 8:** Predictive performance for VTE of four scales.

Scales	TP	FN	TN	FP	Accuracy	Precision	Recall	F1 score
Caprini	87	63	0	150	0.2900	0.3671	0.5800	0.4496
Khorana-2	119	31	17	133	0.4533	0.4722	0.7933	0.5920
Khorana-3	14	136	136	14	0.5000	0.5000	0.0933	0.1573
COMPASS-CAT	9	141	100	50	0.3633	0.1525	0.0600	0.0861

Khorana-2: Khorana score with a threshold of 2.

Khorana-3: Khorana score with a threshold of 3.

In predicting positive events, the Khorana score with a threshold of 3 predicts 136 positive events as negative; while the COMPASS-CAT score predicts 141 positive ones as negative; and the Padua score predicts 115 positive ones as negative. This indicates that these three predictive models may result in the underdiagnosis of high-risk VTE patients with GC/EC, consequently leading to thrombotic events. The Caprini score can identify over half of the high-risk VTE patients with a recall rate of 58.0%, while its predictive performance is moderate. The Khorana score with a threshold of 2 demonstrates the best performance in predicting positive events with a recall rate of 79.3%. The recall rate of this model is lower than that of the GB Model (82.7%) but higher than the positive predictive value of the Simp-GB Model (63.2%).

Regarding the predictive ability of negative samples, the Caprini score shows poor predictive capability by incorrectly identifying all 150 negative cases as positive, which may lead to excessive anticoagulation and increase the iatrogenic risk of bleeding events in patients. The Khorana score with a threshold of 2 incorrectly identifies 88.7% of negative samples as positive. Therefore, if this score is used in clinical practice for the patient population studied by our research group, although the predictive performance of positive samples is relatively good, it would sacrifice the clinical anticoagulation benefits for most low-risk VTE patients. Among the four predictive models, the model with the best predictive performance for negative samples is the COMPASS-CAT score, followed by the Padua score, with specificities of 66.7% and 64.0%, respectively, but both are lower than the GB Model (99.3%) and Simp-GB Model (88.9%) developed in this study.

Through comprehensive evaluation based on accuracy, precision, recall rate, and F1 score, the performance of the GB Model constructed in this study with full feature selection remains optimal, followed by the Simp-GB Model, both of which outperform the four existing predictive models developed thus far.

## 4 Discussion

The incidence of GC and EC in China ranks high globally, significantly impacting the public health level. Tumor-related VTE occurs at a high rate, especially in patients with GC/EC. Additionally, patients with GC/EC are often accompanied by a high risk of upper gastrointestinal bleeding, making early assessment of VTE crucial. Existing tools for assessing VTE are difficult to accurately identify GC/EC patients at high risk of thrombosis.

This study centers on the cohort of GC/EC patients, aiming to thoroughly examine their susceptibility to VTE and the contributing factors. Employing a retrospective study design, we have developed a VTE prediction model for GC/EC patients using machine learning algorithms. This research not only provides clinicians with a robust instrument for the precocious detection of VTE risks in GC/EC patients but also offers valuable data resources for further exploration of thrombogenesis mechanisms and the development of preventive strategies. It is our aspiration that by identifying and intervening in GC/EC patients at elevated VTE risk, we may ameliorate patient prognosis and their quality of life. Specifically, our model assists clinicians in identifying high-risk VTE patients, enabling the customization of more personalized prevention protocols for these individuals. This encompasses, though is not limited to, the adjustment of anticoagulation therapy in terms of timing, intensity, and duration, as well as the judicious employment of mechanical prophylaxis. We anticipate that such precise VTE prevention management will reduce the occurrence of VTE events. Furthermore, the early identification of high-risk patients is instrumental in the optimized allocation of healthcare resources, the minimization of superfluous medical interventions, and the enhancement of patient safety.

Moreover, this study additionally selected four widely used clinical VTE risk assessment models, namely, the Caprini score, Padua score, Khorana score, and COMPASS-CAT score, to externally validate these four risk assessment models using the dataset of this study. The results indicate that these four models have relatively low predictive value for VTE risk in patients with GC/EC. The GB Model developed in this study, as well as the Simp-GB Model, both demonstrate higher predictive value for VTE risk in patients with GC/EC compared to the aforementioned four scoring models, making them more suitable for clinical use in assessing VTE risk in patients with GC/EC.

This study aimed to predict the risk of VTE in patients with GC/EC through the establishment of a predictive model. Essentially, this constituted a valuable exploration into methods for early screening of VTE risk, providing reference for clinical identification and early prevention of thrombosis in patients. However, the study encountered certain limitations in both methodological design and specific implementation processes. As a retrospective study, this research is constrained by the availability of data. Some crucial yet non-routine clinical diagnostic and therapeutic data, such as genetic polymorphisms, thromboelastography, family thrombotic history, among others, were notably absent and difficult to retrieve through the examination of case records. Moreover, chemotherapy regimens often entail the concurrent administration of multiple drugs or undergo alterations in medication over time, both of which are inherently tied to temporal sequences. The algorithms utilized in this study were unable to precisely handle the concomitant use of each medication, ultimately resulting in the incorporation of medication usage as a binary variable, potentially leading to the loss of information related to medication-associated factors. Secondly, the subjects of this study are patients who developed VTE within 6 months of being diagnosed with GC/EC. The prodromal symptoms of VTE are often subtle, and patients may remain unaware of its occurrence without routine screening. Alternatively, they may experience VTE symptoms while receiving treatment at other medical facilities, leading to negative results in our records. Due to limitations in follow-up capacity, this study cannot conduct follow-up investigations for all GC/EC patients in the hospital, potentially resulting in an underestimation of the probability of VTE occurrence in this population. Furthermore, given the relatively low probability of patients experiencing VTE, the dataset exhibits an imbalance between the VTE and non-VTE groups in terms of sample size. Despite employing data sampling algorithms to address this issue, the predictive outcomes of the study may still be susceptible to latent biases. Finally, given the relatively limited volume of data in this study, the generalization capability of the model remains to be further validated. In the future, it is necessary to collect multicenter data or data from different time periods for external validation of the model, in order to comprehensively and objectively evaluate its performance.

To further enhance the clinical applicability of the model, future research will focus on several aspects. Firstly, by incorporating multi-center medical data and data from different time periods to expand the sample size, external validation of the model will be conducted. Based on the validation results, the model will be further optimized to make it more suitable for clinical application. Secondly, prospective application of the model will be employed for risk prediction, and guidelines for the classification and management of thrombosis in GC/EC patients will be developed to achieve personalized treatment. Finally, the model will be subjected to visualization processing, and a webpage or application software will be developed to enable users to apply the predictive model more conveniently.

## Data Availability

The datasets presented in this article are not readily available because, although patient data has been redacted, disclosing this information may still pose potential ethical concerns. Additionally, we utilize an integrated software module for building, training, and deploying various machine learning models. However, due to the restrictions of a specific license agreement, we are unable to provide raw patient data or the GitHub source code. Requests for access to the datasets should be directed to Xingyue Zheng, 1103517296@qq.com.

## Glossary

AdaBoost: Adaptive Boosting;
AI: artificial intelligence
APTR: activated partial thromboplastin ratio
APTT: activated partial thromboplastin time;
ASCO: American Society of Clinical Oncology
AUC: area under the curve
AUCPR: area under the Precision- Recall curve
CA: carbohydrate antigens
CEA: carcinoembryonic antigen
CAT: Cancer Associated Thrombosis
CatBoost: Categorical Boost
CI: confidence interval
CSCO: Chinese Society of Clinical Oncology
CVADs: central venous access devices
DALYs: Disability Adjusted Life Years
D-D: D-dimer
DVT: deep venous thrombosis
EC: Esophageal Cancer
EHRS: electronic health record system
FDP: fibrinogen degradation products
**FI**B: fibrinogen
GBoost: Gradient Boosting
GC: Gastric Cancer
G-CSF: granulocyte colony-stimulating factor
HDL: high-density lipoprotein
HIS: Hospital Information System
hs-CRP: hypersensitive-C reactive protein
hs-TnI: high-sensitivity troponin I
IARC: International Agency for Research on Cancer
ICIs: immune checkpoint inhibitors
INR: international normalized ratio
ISVD: iterative singular value decomposition
KNN: K-Nearest Neighbor
LightGBM: Light Gradient Boosting Machine
LR: logistic regression
NCCN: National Comprehensive Cancer Network
OR: odds ratio
PAI-1: plasminogen activator inhibitor-1
PE: pulmonary embolism
PICO: population, interventions, comparisons, outcomes
RAMs: risk assessment models
RCT: randomized controlled trial
RF: random forest
ROC: receiver operating characteristic
ROS: random over sampling
RR: relative risk
SHAP: shapley additive explanations
SMOTE: synthetic minority oversampling Technique
SMOTEb: borderline-SMOTE
SMOTEN: SMOTE for Nominal
SVM: support vector machine
TF: the release of tissue factor
TT: thrombin time
T5Ms-F: the top five models with full variables
T5Ms-S: the top five models with simplified variables
VEGFR: vascular endothelial growth factor receptor
VTE: venous thromboembolism
WHO: World Health Organization
XGBoost: extreme Gradient Boosting; 5-FU, 5-fluorouracil
