# Supplementary material for: Development and validation of a prediction model for VTE risk in gastric and esophageal cancer patients
Source: Front Pharmacol. 2025 Feb 28;16:1448879. doi: 10.3389/fphar.2025.1448879 (PMC11906997; doi:10.3389/fphar.2025.1448879)
Supplement: Supplementary file 1 [file Supplementaryfile1.docx]

**Appendix A: Ethics review approval**


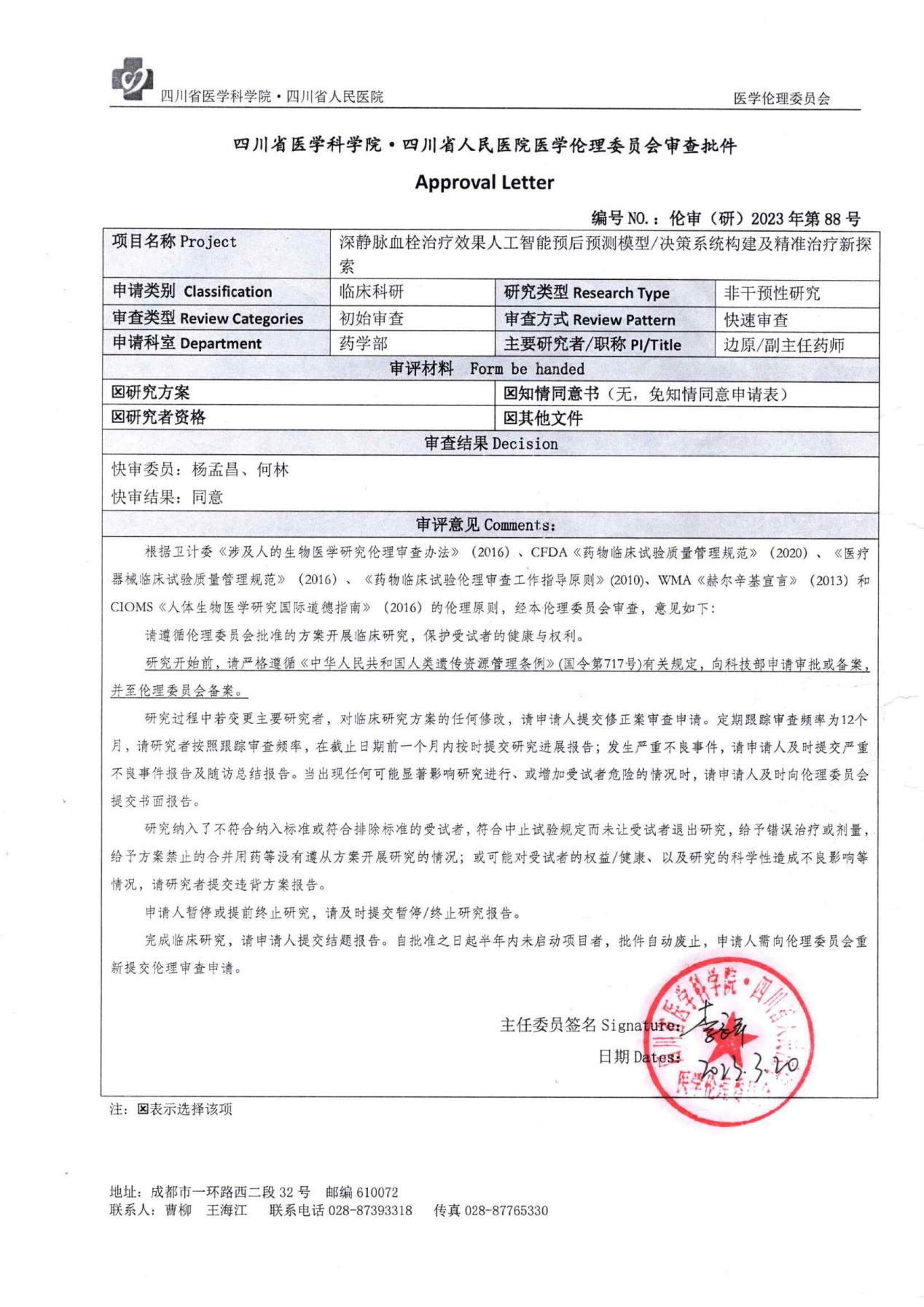


**Appendix B: Performance metrics of the 576 predictive models established with full variables**

| **Data Imputation** | **Data Sampling** | **Feature Selection** | **Algorithm** | **AUC** | **Accuracy** | **Precision** | **Recall** | **F1 Score** |
| --- | --- | --- | --- | --- | --- | --- | --- | --- |
| 0 | 0 | 0 | 0 | 0.7359 | 0.6745 | 0.6757 | 0.6711 | 0.6734 |
| 0 | 0 | 0 | 1 | 0.7223 | 0.6477 | 0.6964 | 0.5235 | 0.5977 |
| 0 | 0 | 0 | 2 | 0.6486 | 0.6477 | 0.6618 | 0.6040 | 0.6316 |
| 0 | 0 | 0 | 3 | 0.8107 | 0.5302 | 0.8462 | 0.0738 | 0.1358 |
| 0 | 0 | 0 | 4 | 0.7289 | 0.5369 | 0.6897 | 0.1342 | 0.2247 |
| 0 | 0 | 0 | 5 | 0.6798 | 0.6242 | 0.7033 | 0.4295 | 0.5333 |
| 0 | 0 | 0 | 6 | 0.7564 | 0.5302 | 0.8000 | 0.0805 | 0.1463 |
| 0 | 0 | 0 | 7 | 0.7429 | 0.5235 | 0.8182 | 0.0604 | 0.1125 |
| 0 | 0 | 0 | 8 | 0.7033 | 0.6107 | 0.7705 | 0.3154 | 0.4476 |
| 0 | 0 | 1 | 0 | 0.7310 | 0.6846 | 0.6897 | 0.6711 | 0.6803 |
| 0 | 0 | 1 | 1 | 0.7031 | 0.6174 | 0.6667 | 0.4698 | 0.5512 |
| 0 | 0 | 1 | 2 | 0.6860 | 0.6007 | 0.6250 | 0.5034 | 0.5576 |
| 0 | 0 | 1 | 3 | 0.7729 | 0.5336 | 0.9167 | 0.0738 | 0.1366 |
| 0 | 0 | 1 | 4 | 0.7222 | 0.5906 | 0.8462 | 0.2215 | 0.3511 |
| 0 | 0 | 1 | 5 | 0.6108 | 0.6409 | 0.6780 | 0.5369 | 0.5993 |
| 0 | 0 | 1 | 6 | 0.7600 | 0.6074 | 0.9000 | 0.2416 | 0.3810 |
| 0 | 0 | 1 | 7 | 0.7222 | 0.6107 | 0.9231 | 0.2416 | 0.3830 |
| 0 | 0 | 1 | 8 | 0.6835 | 0.6141 | 0.7361 | 0.3557 | 0.4796 |
| 0 | 0 | 2 | 0 | 0.5699 | 0.5034 | 0.5035 | 0.4832 | 0.4932 |
| 0 | 0 | 2 | 1 | 0.5665 | 0.5436 | 0.5765 | 0.3289 | 0.4188 |
| 0 | 0 | 2 | 2 | 0.4243 | 0.4664 | 0.2727 | 0.0403 | 0.0702 |
| 0 | 0 | 2 | 3 | 0.5127 | 0.5034 | 0.5060 | 0.2819 | 0.3621 |
| 0 | 0 | 2 | 4 | 0.5105 | 0.5101 | 0.5185 | 0.2819 | 0.3652 |
| 0 | 0 | 2 | 5 | 0.6142 | 0.5738 | 0.5786 | 0.5436 | 0.5606 |
| 0 | 0 | 2 | 6 | 0.5284 | 0.4832 | 0.4675 | 0.2416 | 0.3186 |
| 0 | 0 | 2 | 7 | 0.4830 | 0.5201 | 0.5385 | 0.2819 | 0.3700 |
| 0 | 0 | 2 | 8 | 0.5514 | 0.5906 | 0.6134 | 0.4899 | 0.5448 |
| 0 | 0 | 3 | 0 | 0.7359 | 0.6745 | 0.6757 | 0.6711 | 0.6734 |
| 0 | 0 | 3 | 1 | 0.7223 | 0.6477 | 0.6964 | 0.5235 | 0.5977 |
| 0 | 0 | 3 | 2 | 0.6486 | 0.6477 | 0.6618 | 0.6040 | 0.6316 |
| 0 | 0 | 3 | 3 | 0.8107 | 0.5302 | 0.8462 | 0.0738 | 0.1358 |
| 0 | 0 | 3 | 4 | 0.7289 | 0.5369 | 0.6897 | 0.1342 | 0.2247 |
| 0 | 0 | 3 | 5 | 0.6798 | 0.6242 | 0.7033 | 0.4295 | 0.5333 |
| 0 | 0 | 3 | 6 | 0.7564 | 0.5302 | 0.8000 | 0.0805 | 0.1463 |
| 0 | 0 | 3 | 7 | 0.7429 | 0.5235 | 0.8182 | 0.0604 | 0.1125 |
| 0 | 0 | 3 | 8 | 0.7033 | 0.6107 | 0.7705 | 0.3154 | 0.4476 |
| 0 | 1 | 0 | 0 | 0.8873 | 0.8423 | 0.8696 | 0.8054 | 0.8362 |
| 0 | 1 | 0 | 1 | 0.8787 | 0.7987 | 0.8678 | 0.7047 | 0.7778 |
| 0 | 1 | 0 | 2 | 0.7411 | 0.6913 | 0.6492 | 0.8322 | 0.7294 |
| 0 | 1 | 0 | 3 | 0.9423 | 0.8523 | 0.9817 | 0.7181 | 0.8295 |
| 0 | 1 | 0 | 4 | 0.9442 | 0.8725 | 0.9440 | 0.7919 | 0.8613 |
| 0 | 1 | 0 | 5 | 0.8773 | 0.8389 | 0.8855 | 0.7785 | 0.8286 |
| 0 | 1 | 0 | 6 | 0.9302 | 0.8658 | 0.9658 | 0.7584 | 0.8496 |
| 0 | 1 | 0 | 7 | 0.9447 | 0.8658 | 0.9658 | 0.7584 | 0.8496 |
| 0 | 1 | 0 | 8 | 0.9560 | 0.9060 | 0.9481 | 0.8591 | 0.9014 |
| 0 | 1 | 1 | 0 | 0.8780 | 0.8221 | 0.8692 | 0.7584 | 0.8100 |
| 0 | 1 | 1 | 1 | 0.8746 | 0.7953 | 0.8793 | 0.6846 | 0.7698 |
| 0 | 1 | 1 | 2 | 0.7933 | 0.7282 | 0.7099 | 0.7718 | 0.7395 |
| 0 | 1 | 1 | 3 | 0.9439 | 0.8658 | 0.9580 | 0.7651 | 0.8507 |
| 0 | 1 | 1 | 4 | 0.9129 | 0.8557 | 0.9206 | 0.7785 | 0.8436 |
| 0 | 1 | 1 | 5 | 0.9225 | 0.8758 | 0.8889 | 0.8591 | 0.8737 |
| 0 | 1 | 1 | 6 | 0.9063 | 0.8423 | 0.9474 | 0.7248 | 0.8213 |
| 0 | 1 | 1 | 7 | 0.9298 | 0.8456 | 0.9402 | 0.7383 | 0.8271 |
| 0 | 1 | 1 | 8 | 0.9380 | 0.8658 | 0.9225 | 0.7987 | 0.8561 |
| 0 | 1 | 2 | 0 | 0.7255 | 0.7181 | 0.6650 | 0.8792 | 0.7572 |
| 0 | 1 | 2 | 1 | 0.7578 | 0.7383 | 0.7006 | 0.8322 | 0.7607 |
| 0 | 1 | 2 | 2 | 0.5789 | 0.5268 | 0.6176 | 0.1409 | 0.2295 |
| 0 | 1 | 2 | 3 | 0.6913 | 0.6980 | 0.6725 | 0.7718 | 0.7188 |
| 0 | 1 | 2 | 4 | 0.6906 | 0.6913 | 0.6667 | 0.7651 | 0.7125 |
| 0 | 1 | 2 | 5 | 0.7259 | 0.7081 | 0.6566 | 0.8725 | 0.7493 |
| 0 | 1 | 2 | 6 | 0.7316 | 0.7114 | 0.6842 | 0.7852 | 0.7313 |
| 0 | 1 | 2 | 7 | 0.7096 | 0.7215 | 0.6875 | 0.8121 | 0.7446 |
| 0 | 1 | 2 | 8 | 0.7335 | 0.7349 | 0.7011 | 0.8188 | 0.7554 |
| 0 | 1 | 3 | 0 | 0.8873 | 0.8423 | 0.8696 | 0.8054 | 0.8362 |
| 0 | 1 | 3 | 1 | 0.8787 | 0.7987 | 0.8678 | 0.7047 | 0.7778 |
| 0 | 1 | 3 | 2 | 0.7411 | 0.6913 | 0.6492 | 0.8322 | 0.7294 |
| 0 | 1 | 3 | 3 | 0.9423 | 0.8523 | 0.9817 | 0.7181 | 0.8295 |
| 0 | 1 | 3 | 4 | 0.9442 | 0.8725 | 0.9440 | 0.7919 | 0.8613 |
| 0 | 1 | 3 | 5 | 0.8773 | 0.8389 | 0.8855 | 0.7785 | 0.8286 |
| 0 | 1 | 3 | 6 | 0.9302 | 0.8658 | 0.9658 | 0.7584 | 0.8496 |
| 0 | 1 | 3 | 7 | 0.9447 | 0.8658 | 0.9658 | 0.7584 | 0.8496 |
| 0 | 1 | 3 | 8 | 0.9560 | 0.9060 | 0.9481 | 0.8591 | 0.9014 |
| 0 | 2 | 0 | 0 | 0.9368 | 0.8859 | 0.8912 | 0.8792 | 0.8851 |
| 0 | 2 | 0 | 1 | 0.9139 | 0.8423 | 0.8864 | 0.7852 | 0.8327 |
| 0 | 2 | 0 | 2 | 0.7711 | 0.6745 | 0.6512 | 0.7517 | 0.6978 |
| 0 | 2 | 0 | 3 | 0.9541 | 0.8624 | 0.9737 | 0.7450 | 0.8441 |
| 0 | 2 | 0 | 4 | 0.9462 | 0.8826 | 0.9597 | 0.7987 | 0.8718 |
| 0 | 2 | 0 | 5 | 0.8806 | 0.8658 | 0.9291 | 0.7919 | 0.8551 |
| 0 | 2 | 0 | 6 | 0.9457 | 0.8725 | 0.9512 | 0.7852 | 0.8603 |
| 0 | 2 | 0 | 7 | 0.9534 | 0.8893 | 0.9754 | 0.7987 | 0.8782 |
| 0 | 2 | 0 | 8 | 0.9559 | 0.9027 | 0.9348 | 0.8658 | 0.8990 |
| 0 | 2 | 1 | 0 | 0.9273 | 0.8691 | 0.8873 | 0.8456 | 0.8660 |
| 0 | 2 | 1 | 1 | 0.8991 | 0.8087 | 0.8538 | 0.7450 | 0.7957 |
| 0 | 2 | 1 | 2 | 0.8022 | 0.7416 | 0.7400 | 0.7450 | 0.7425 |
| 0 | 2 | 1 | 3 | 0.9374 | 0.8591 | 0.9496 | 0.7584 | 0.8433 |
| 0 | 2 | 1 | 4 | 0.9237 | 0.8624 | 0.9500 | 0.7651 | 0.8476 |
| 0 | 2 | 1 | 5 | 0.9379 | 0.8792 | 0.8897 | 0.8658 | 0.8776 |
| 0 | 2 | 1 | 6 | 0.9214 | 0.8624 | 0.9219 | 0.7919 | 0.8520 |
| 0 | 2 | 1 | 7 | 0.9444 | 0.8591 | 0.9213 | 0.7852 | 0.8478 |
| 0 | 2 | 1 | 8 | 0.9477 | 0.8826 | 0.9014 | 0.8591 | 0.8797 |
| 0 | 2 | 2 | 0 | 0.6946 | 0.6812 | 0.6484 | 0.7919 | 0.7130 |
| 0 | 2 | 2 | 1 | 0.7242 | 0.7013 | 0.6852 | 0.7450 | 0.7138 |
| 0 | 2 | 2 | 2 | 0.5543 | 0.4698 | 0.2857 | 0.0403 | 0.0706 |
| 0 | 2 | 2 | 3 | 0.6455 | 0.6074 | 0.6081 | 0.6040 | 0.6061 |
| 0 | 2 | 2 | 4 | 0.6366 | 0.6342 | 0.6190 | 0.6980 | 0.6562 |
| 0 | 2 | 2 | 5 | 0.6874 | 0.6846 | 0.6486 | 0.8054 | 0.7186 |
| 0 | 2 | 2 | 6 | 0.6548 | 0.6577 | 0.6460 | 0.6980 | 0.6710 |
| 0 | 2 | 2 | 7 | 0.6640 | 0.6309 | 0.6242 | 0.6577 | 0.6405 |
| 0 | 2 | 2 | 8 | 0.7220 | 0.6946 | 0.6768 | 0.7450 | 0.7093 |
| 0 | 2 | 3 | 0 | 0.9368 | 0.8859 | 0.8912 | 0.8792 | 0.8851 |
| 0 | 2 | 3 | 1 | 0.9139 | 0.8423 | 0.8864 | 0.7852 | 0.8327 |
| 0 | 2 | 3 | 2 | 0.7711 | 0.6745 | 0.6512 | 0.7517 | 0.6978 |
| 0 | 2 | 3 | 3 | 0.9541 | 0.8624 | 0.9737 | 0.7450 | 0.8441 |
| 0 | 2 | 3 | 4 | 0.9462 | 0.8826 | 0.9597 | 0.7987 | 0.8718 |
| 0 | 2 | 3 | 5 | 0.8806 | 0.8658 | 0.9291 | 0.7919 | 0.8551 |
| 0 | 2 | 3 | 6 | 0.9457 | 0.8725 | 0.9512 | 0.7852 | 0.8603 |
| 0 | 2 | 3 | 7 | 0.9534 | 0.8893 | 0.9754 | 0.7987 | 0.8782 |
| 0 | 2 | 3 | 8 | 0.9559 | 0.9027 | 0.9348 | 0.8658 | 0.8990 |
| 0 | 3 | 0 | 0 | 0.9338 | 0.8960 | 0.9097 | 0.8792 | 0.8942 |
| 0 | 3 | 0 | 1 | 0.9431 | 0.9161 | 0.9844 | 0.8456 | 0.9097 |
| 0 | 3 | 0 | 2 | 0.9144 | 0.8691 | 0.8667 | 0.8725 | 0.8696 |
| 0 | 3 | 0 | 3 | 0.9620 | 0.9161 | 0.9921 | 0.8389 | 0.9091 |
| 0 | 3 | 0 | 4 | 0.9409 | 0.9027 | 0.9478 | 0.8523 | 0.8975 |
| 0 | 3 | 0 | 5 | 0.9382 | 0.9060 | 0.9172 | 0.8926 | 0.9048 |
| 0 | 3 | 0 | 6 | 0.9397 | 0.8960 | 0.9836 | 0.8054 | 0.8856 |
| 0 | 3 | 0 | 7 | 0.9462 | 0.9195 | 0.9845 | 0.8523 | 0.9137 |
| 0 | 3 | 0 | 8 | 0.9449 | 0.9128 | 0.9624 | 0.8591 | 0.9078 |
| 0 | 3 | 1 | 0 | 0.9260 | 0.8993 | 0.9161 | 0.8792 | 0.8973 |
| 0 | 3 | 1 | 1 | 0.9468 | 0.9128 | 0.9767 | 0.8456 | 0.9065 |
| 0 | 3 | 1 | 2 | 0.9252 | 0.8960 | 0.9097 | 0.8792 | 0.8942 |
| 0 | 3 | 1 | 3 | 0.9431 | 0.9161 | 0.9844 | 0.8456 | 0.9097 |
| 0 | 3 | 1 | 4 | 0.9296 | 0.9060 | 0.9416 | 0.8658 | 0.9021 |
| 0 | 3 | 1 | 5 | 0.9235 | 0.8926 | 0.9091 | 0.8725 | 0.8904 |
| 0 | 3 | 1 | 6 | 0.9350 | 0.9094 | 0.9552 | 0.8591 | 0.9046 |
| 0 | 3 | 1 | 7 | 0.9354 | 0.9228 | 0.9773 | 0.8658 | 0.9181 |
| 0 | 3 | 1 | 8 | 0.9273 | 0.9094 | 0.9485 | 0.8658 | 0.9053 |
| 0 | 3 | 2 | 0 | 0.7694 | 0.6946 | 0.6526 | 0.8322 | 0.7316 |
| 0 | 3 | 2 | 1 | 0.6926 | 0.6477 | 0.6429 | 0.6644 | 0.6535 |
| 0 | 3 | 2 | 2 | 0.3825 | 0.5000 | 0.5000 | 0.0336 | 0.0629 |
| 0 | 3 | 2 | 3 | 0.6859 | 0.6409 | 0.6400 | 0.6443 | 0.6421 |
| 0 | 3 | 2 | 4 | 0.6634 | 0.6141 | 0.6104 | 0.6309 | 0.6205 |
| 0 | 3 | 2 | 5 | 0.7655 | 0.6980 | 0.6612 | 0.8121 | 0.7289 |
| 0 | 3 | 2 | 6 | 0.6504 | 0.5872 | 0.6016 | 0.5168 | 0.5560 |
| 0 | 3 | 2 | 7 | 0.6991 | 0.6141 | 0.6133 | 0.6174 | 0.6154 |
| 0 | 3 | 2 | 8 | 0.7322 | 0.6376 | 0.6185 | 0.7181 | 0.6646 |
| 0 | 3 | 3 | 0 | 0.9338 | 0.8960 | 0.9097 | 0.8792 | 0.8942 |
| 0 | 3 | 3 | 1 | 0.9431 | 0.9161 | 0.9844 | 0.8456 | 0.9097 |
| 0 | 3 | 3 | 2 | 0.9144 | 0.8691 | 0.8667 | 0.8725 | 0.8696 |
| 0 | 3 | 3 | 3 | 0.9620 | 0.9161 | 0.9921 | 0.8389 | 0.9091 |
| 0 | 3 | 3 | 4 | 0.9409 | 0.9027 | 0.9478 | 0.8523 | 0.8975 |
| 0 | 3 | 3 | 5 | 0.9382 | 0.9060 | 0.9172 | 0.8926 | 0.9048 |
| 0 | 3 | 3 | 6 | 0.9397 | 0.8960 | 0.9836 | 0.8054 | 0.8856 |
| 0 | 3 | 3 | 7 | 0.9462 | 0.9195 | 0.9845 | 0.8523 | 0.9137 |
| 0 | 3 | 3 | 8 | 0.9449 | 0.9128 | 0.9624 | 0.8591 | 0.9078 |
| 1 | 0 | 0 | 0 | 0.7694 | 0.7033 | 0.7226 | 0.6600 | 0.6899 |
| 1 | 0 | 0 | 1 | 0.7676 | 0.6433 | 0.6972 | 0.5067 | 0.5869 |
| 1 | 0 | 0 | 2 | 0.6606 | 0.6000 | 0.6389 | 0.4600 | 0.5349 |
| 1 | 0 | 0 | 3 | 0.7518 | 0.5200 | 1.0000 | 0.0400 | 0.0769 |
| 1 | 0 | 0 | 4 | 0.6861 | 0.6067 | 0.8810 | 0.2467 | 0.3854 |
| 1 | 0 | 0 | 5 | 0.7141 | 0.6433 | 0.7048 | 0.4933 | 0.5804 |
| 1 | 0 | 0 | 6 | 0.7116 | 0.6067 | 0.9444 | 0.2267 | 0.3656 |
| 1 | 0 | 0 | 7 | 0.7436 | 0.6333 | 1.0000 | 0.2667 | 0.4211 |
| 1 | 0 | 0 | 8 | 0.7395 | 0.7133 | 0.9103 | 0.4733 | 0.6228 |
| 1 | 0 | 1 | 0 | 0.7682 | 0.6567 | 0.6942 | 0.5600 | 0.6199 |
| 1 | 0 | 1 | 1 | 0.7446 | 0.6033 | 0.6566 | 0.4333 | 0.5221 |
| 1 | 0 | 1 | 2 | 0.6248 | 0.6100 | 0.6410 | 0.5000 | 0.5618 |
| 1 | 0 | 1 | 3 | 0.7820 | 0.5333 | 0.9167 | 0.0733 | 0.1358 |
| 1 | 0 | 1 | 4 | 0.7092 | 0.5367 | 0.7391 | 0.1133 | 0.1965 |
| 1 | 0 | 1 | 5 | 0.6980 | 0.6700 | 0.7073 | 0.5800 | 0.6374 |
| 1 | 0 | 1 | 6 | 0.6813 | 0.5133 | 0.6111 | 0.0733 | 0.1310 |
| 1 | 0 | 1 | 7 | 0.7044 | 0.5300 | 0.8462 | 0.0733 | 0.1350 |
| 1 | 0 | 1 | 8 | 0.7119 | 0.6000 | 0.7143 | 0.3333 | 0.4545 |
| 1 | 0 | 2 | 0 | 0.6424 | 0.6200 | 0.6200 | 0.6200 | 0.6200 |
| 1 | 0 | 2 | 1 | 0.6064 | 0.6033 | 0.6281 | 0.5067 | 0.5609 |
| 1 | 0 | 2 | 2 | 0.6324 | 0.6000 | 0.7679 | 0.2867 | 0.4175 |
| 1 | 0 | 2 | 3 | 0.6268 | 0.5933 | 0.6186 | 0.4867 | 0.5448 |
| 1 | 0 | 2 | 4 | 0.5827 | 0.6100 | 0.6854 | 0.4067 | 0.5105 |
| 1 | 0 | 2 | 5 | 0.6711 | 0.6267 | 0.6357 | 0.5933 | 0.6138 |
| 1 | 0 | 2 | 6 | 0.6136 | 0.6000 | 0.6667 | 0.4000 | 0.5000 |
| 1 | 0 | 2 | 7 | 0.5474 | 0.5600 | 0.6184 | 0.3133 | 0.4159 |
| 1 | 0 | 2 | 8 | 0.6089 | 0.6200 | 0.6698 | 0.4733 | 0.5547 |
| 1 | 0 | 3 | 0 | 0.7694 | 0.7033 | 0.7226 | 0.6600 | 0.6899 |
| 1 | 0 | 3 | 1 | 0.7676 | 0.6433 | 0.6972 | 0.5067 | 0.5869 |
| 1 | 0 | 3 | 2 | 0.6606 | 0.6000 | 0.6389 | 0.4600 | 0.5349 |
| 1 | 0 | 3 | 3 | 0.7518 | 0.5200 | 1.0000 | 0.0400 | 0.0769 |
| 1 | 0 | 3 | 4 | 0.6861 | 0.6067 | 0.8810 | 0.2467 | 0.3854 |
| 1 | 0 | 3 | 5 | 0.7141 | 0.6433 | 0.7048 | 0.4933 | 0.5804 |
| 1 | 0 | 3 | 6 | 0.7116 | 0.6067 | 0.9444 | 0.2267 | 0.3656 |
| 1 | 0 | 3 | 7 | 0.7436 | 0.6333 | 1.0000 | 0.2667 | 0.4211 |
| 1 | 0 | 3 | 8 | 0.7395 | 0.7133 | 0.9103 | 0.4733 | 0.6228 |
| 1 | 1 | 0 | 0 | 0.7344 | 0.6800 | 0.7143 | 0.6000 | 0.6522 |
| 1 | 1 | 0 | 1 | 0.7288 | 0.6333 | 0.7083 | 0.4533 | 0.5528 |
| 1 | 1 | 0 | 2 | 0.6962 | 0.6933 | 0.6790 | 0.7333 | 0.7051 |
| 1 | 1 | 0 | 3 | 0.9474 | 0.8100 | 0.9697 | 0.6400 | 0.7711 |
| 1 | 1 | 0 | 4 | 0.9420 | 0.8367 | 0.9903 | 0.6800 | 0.8063 |
| 1 | 1 | 0 | 5 | 0.9388 | 0.8467 | 0.8824 | 0.8000 | 0.8392 |
| 1 | 1 | 0 | 6 | 0.9609 | 0.8833 | 0.9915 | 0.7733 | 0.8689 |
| 1 | 1 | 0 | 7 | 0.9516 | 0.8700 | 0.9826 | 0.7533 | 0.8528 |
| 1 | 1 | 0 | 8 | 0.9646 | 0.9100 | 0.9920 | 0.8267 | 0.9018 |
| 1 | 1 | 1 | 0 | 0.7368 | 0.6933 | 0.7302 | 0.6133 | 0.6667 |
| 1 | 1 | 1 | 1 | 0.7591 | 0.6867 | 0.7642 | 0.5400 | 0.6328 |
| 1 | 1 | 1 | 2 | 0.7048 | 0.6400 | 0.6419 | 0.6333 | 0.6376 |
| 1 | 1 | 1 | 3 | 0.9482 | 0.8633 | 0.9910 | 0.7333 | 0.8429 |
| 1 | 1 | 1 | 4 | 0.9066 | 0.8333 | 0.9386 | 0.7133 | 0.8106 |
| 1 | 1 | 1 | 5 | 0.9140 | 0.8233 | 0.8540 | 0.7800 | 0.8153 |
| 1 | 1 | 1 | 6 | 0.9431 | 0.8500 | 0.9565 | 0.7333 | 0.8302 |
| 1 | 1 | 1 | 7 | 0.9299 | 0.8533 | 0.9649 | 0.7333 | 0.8333 |
| 1 | 1 | 1 | 8 | 0.9447 | 0.8967 | 0.9760 | 0.8133 | 0.8873 |
| 1 | 1 | 2 | 0 | 0.4715 | 0.4467 | 0.3974 | 0.2067 | 0.2719 |
| 1 | 1 | 2 | 1 | 0.6730 | 0.5433 | 0.6066 | 0.2467 | 0.3507 |
| 1 | 1 | 2 | 2 | 0.7241 | 0.6500 | 0.7228 | 0.4867 | 0.5817 |
| 1 | 1 | 2 | 3 | 0.7781 | 0.7333 | 0.9070 | 0.5200 | 0.6610 |
| 1 | 1 | 2 | 4 | 0.7923 | 0.7333 | 0.8977 | 0.5267 | 0.6639 |
| 1 | 1 | 2 | 5 | 0.8135 | 0.7567 | 0.8969 | 0.5800 | 0.7045 |
| 1 | 1 | 2 | 6 | 0.7962 | 0.7467 | 0.9022 | 0.5533 | 0.6860 |
| 1 | 1 | 2 | 7 | 0.7998 | 0.7500 | 0.9213 | 0.5467 | 0.6862 |
| 1 | 1 | 2 | 8 | 0.8145 | 0.7600 | 0.9239 | 0.5667 | 0.7025 |
| 1 | 1 | 3 | 0 | 0.7344 | 0.6800 | 0.7143 | 0.6000 | 0.6522 |
| 1 | 1 | 3 | 1 | 0.7288 | 0.6333 | 0.7083 | 0.4533 | 0.5528 |
| 1 | 1 | 3 | 2 | 0.6962 | 0.6933 | 0.6790 | 0.7333 | 0.7051 |
| 1 | 1 | 3 | 3 | 0.9474 | 0.8100 | 0.9697 | 0.6400 | 0.7711 |
| 1 | 1 | 3 | 4 | 0.9420 | 0.8367 | 0.9903 | 0.6800 | 0.8063 |
| 1 | 1 | 3 | 5 | 0.9388 | 0.8467 | 0.8824 | 0.8000 | 0.8392 |
| 1 | 1 | 3 | 6 | 0.9609 | 0.8833 | 0.9915 | 0.7733 | 0.8689 |
| 1 | 1 | 3 | 7 | 0.9516 | 0.8700 | 0.9826 | 0.7533 | 0.8528 |
| 1 | 1 | 3 | 8 | 0.9646 | 0.9100 | 0.9920 | 0.8267 | 0.9018 |
| 1 | 2 | 0 | 0 | 0.7558 | 0.7200 | 0.7538 | 0.6533 | 0.7000 |
| 1 | 2 | 0 | 1 | 0.7562 | 0.6567 | 0.7423 | 0.4800 | 0.5830 |
| 1 | 2 | 0 | 2 | 0.6938 | 0.6633 | 0.6690 | 0.6467 | 0.6576 |
| 1 | 2 | 0 | 3 | 0.9416 | 0.8367 | 0.9903 | 0.6800 | 0.8063 |
| 1 | 2 | 0 | 4 | 0.9420 | 0.8600 | 0.9821 | 0.7333 | 0.8397 |
| 1 | 2 | 0 | 5 | 0.9256 | 0.8300 | 0.8613 | 0.7867 | 0.8223 |
| 1 | 2 | 0 | 6 | 0.9515 | 0.8833 | 0.9915 | 0.7733 | 0.8689 |
| 1 | 2 | 0 | 7 | 0.9467 | 0.8633 | 0.9910 | 0.7333 | 0.8429 |
| 1 | 2 | 0 | 8 | 0.9642 | 0.9200 | 0.9922 | 0.8467 | 0.9137 |
| 1 | 2 | 1 | 0 | 0.7584 | 0.7367 | 0.7591 | 0.6933 | 0.7247 |
| 1 | 2 | 1 | 1 | 0.7900 | 0.6600 | 0.7353 | 0.5000 | 0.5952 |
| 1 | 2 | 1 | 2 | 0.6847 | 0.6433 | 0.6483 | 0.6267 | 0.6373 |
| 1 | 2 | 1 | 3 | 0.9458 | 0.8333 | 0.9808 | 0.6800 | 0.8031 |
| 1 | 2 | 1 | 4 | 0.9094 | 0.8400 | 0.9322 | 0.7333 | 0.8209 |
| 1 | 2 | 1 | 5 | 0.8827 | 0.8267 | 0.8657 | 0.7733 | 0.8169 |
| 1 | 2 | 1 | 6 | 0.9262 | 0.8367 | 0.9720 | 0.6933 | 0.8093 |
| 1 | 2 | 1 | 7 | 0.9281 | 0.8367 | 0.9633 | 0.7000 | 0.8108 |
| 1 | 2 | 1 | 8 | 0.9413 | 0.8833 | 0.9600 | 0.8000 | 0.8727 |
| 1 | 2 | 2 | 0 | 0.5401 | 0.5433 | 0.5442 | 0.5333 | 0.5387 |
| 1 | 2 | 2 | 1 | 0.7163 | 0.6400 | 0.6944 | 0.5000 | 0.5814 |
| 1 | 2 | 2 | 2 | 0.7256 | 0.6900 | 0.8904 | 0.4333 | 0.5830 |
| 1 | 2 | 2 | 3 | 0.8073 | 0.7733 | 0.9556 | 0.5733 | 0.7167 |
| 1 | 2 | 2 | 4 | 0.8252 | 0.7867 | 0.9674 | 0.5933 | 0.7355 |
| 1 | 2 | 2 | 5 | 0.7921 | 0.7567 | 0.9425 | 0.5467 | 0.6920 |
| 1 | 2 | 2 | 6 | 0.8128 | 0.7700 | 0.9355 | 0.5800 | 0.7160 |
| 1 | 2 | 2 | 7 | 0.8124 | 0.7233 | 0.8018 | 0.5933 | 0.6820 |
| 1 | 2 | 2 | 8 | 0.8215 | 0.7233 | 0.7965 | 0.6000 | 0.6844 |
| 1 | 2 | 3 | 0 | 0.7558 | 0.7200 | 0.7538 | 0.6533 | 0.7000 |
| 1 | 2 | 3 | 1 | 0.7562 | 0.6567 | 0.7423 | 0.4800 | 0.5830 |
| 1 | 2 | 3 | 2 | 0.6938 | 0.6633 | 0.6690 | 0.6467 | 0.6576 |
| 1 | 2 | 3 | 3 | 0.9416 | 0.8367 | 0.9903 | 0.6800 | 0.8063 |
| 1 | 2 | 3 | 4 | 0.9420 | 0.8600 | 0.9821 | 0.7333 | 0.8397 |
| 1 | 2 | 3 | 5 | 0.9256 | 0.8300 | 0.8613 | 0.7867 | 0.8223 |
| 1 | 2 | 3 | 6 | 0.9515 | 0.8833 | 0.9915 | 0.7733 | 0.8689 |
| 1 | 2 | 3 | 7 | 0.9467 | 0.8633 | 0.9910 | 0.7333 | 0.8429 |
| 1 | 2 | 3 | 8 | 0.9642 | 0.9200 | 0.9922 | 0.8467 | 0.9137 |
| 1 | 3 | 0 | 0 | 0.9220 | 0.8867 | 0.9394 | 0.8267 | 0.8794 |
| 1 | 3 | 0 | 1 | 0.9413 | 0.8933 | 0.9917 | 0.7933 | 0.8815 |
| 1 | 3 | 0 | 2 | 0.9387 | 0.9033 | 0.9353 | 0.8667 | 0.8997 |
| 1 | 3 | 0 | 3 | 0.9265 | 0.8000 | 1.0000 | 0.6000 | 0.7500 |
| 1 | 3 | 0 | 4 | 0.8810 | 0.8067 | 1.0000 | 0.6133 | 0.7603 |
| 1 | 3 | 0 | 5 | 0.8436 | 0.7233 | 0.8681 | 0.5267 | 0.6556 |
| 1 | 3 | 0 | 6 | 0.8724 | 0.7733 | 0.9767 | 0.5600 | 0.7119 |
| 1 | 3 | 0 | 7 | 0.9452 | 0.8667 | 1.0000 | 0.7333 | 0.8462 |
| 1 | 3 | 0 | 8 | 0.9077 | 0.8467 | 0.9561 | 0.7267 | 0.8258 |
| 1 | 3 | 1 | 0 | 0.9196 | 0.8867 | 0.9462 | 0.8200 | 0.8786 |
| 1 | 3 | 1 | 1 | 0.9377 | 0.8500 | 0.9907 | 0.7067 | 0.8249 |
| 1 | 3 | 1 | 2 | 0.9276 | 0.8900 | 0.9606 | 0.8133 | 0.8809 |
| 1 | 3 | 1 | 3 | 0.8743 | 0.8467 | 1.0000 | 0.6933 | 0.8189 |
| 1 | 3 | 1 | 4 | 0.8600 | 0.8200 | 0.9528 | 0.6733 | 0.7891 |
| 1 | 3 | 1 | 5 | 0.8080 | 0.8300 | 0.9304 | 0.7133 | 0.8075 |
| 1 | 3 | 1 | 6 | 0.8412 | 0.8267 | 0.9623 | 0.6800 | 0.7969 |
| 1 | 3 | 1 | 7 | 0.9035 | 0.8533 | 0.9907 | 0.7133 | 0.8295 |
| 1 | 3 | 1 | 8 | 0.8555 | 0.8433 | 0.9328 | 0.7400 | 0.8253 |
| 1 | 3 | 2 | 0 | 0.8059 | 0.7433 | 0.7039 | 0.8400 | 0.7660 |
| 1 | 3 | 2 | 1 | 0.8119 | 0.7433 | 0.7212 | 0.7933 | 0.7556 |
| 1 | 3 | 2 | 2 | 0.5320 | 0.5400 | 0.6579 | 0.1667 | 0.2660 |
| 1 | 3 | 2 | 3 | 0.7847 | 0.7333 | 0.7188 | 0.7667 | 0.7419 |
| 1 | 3 | 2 | 4 | 0.7750 | 0.7200 | 0.7063 | 0.7533 | 0.7290 |
| 1 | 3 | 2 | 5 | 0.7897 | 0.7300 | 0.6885 | 0.8400 | 0.7568 |
| 1 | 3 | 2 | 6 | 0.7927 | 0.7367 | 0.7261 | 0.7600 | 0.7427 |
| 1 | 3 | 2 | 7 | 0.8047 | 0.7833 | 0.7607 | 0.8267 | 0.7923 |
| 1 | 3 | 2 | 8 | 0.8077 | 0.7567 | 0.7251 | 0.8267 | 0.7726 |
| 1 | 3 | 3 | 0 | 0.9220 | 0.8867 | 0.9394 | 0.8267 | 0.8794 |
| 1 | 3 | 3 | 1 | 0.9413 | 0.8933 | 0.9917 | 0.7933 | 0.8815 |
| 1 | 3 | 3 | 2 | 0.9387 | 0.9033 | 0.9353 | 0.8667 | 0.8997 |
| 1 | 3 | 3 | 3 | 0.9265 | 0.8000 | 1.0000 | 0.6000 | 0.7500 |
| 1 | 3 | 3 | 4 | 0.8810 | 0.8067 | 1.0000 | 0.6133 | 0.7603 |
| 1 | 3 | 3 | 5 | 0.8436 | 0.7233 | 0.8681 | 0.5267 | 0.6556 |
| 1 | 3 | 3 | 6 | 0.8724 | 0.7733 | 0.9767 | 0.5600 | 0.7119 |
| 1 | 3 | 3 | 7 | 0.9452 | 0.8667 | 1.0000 | 0.7333 | 0.8462 |
| 1 | 3 | 3 | 8 | 0.9077 | 0.8467 | 0.9561 | 0.7267 | 0.8258 |
| 2 | 0 | 0 | 0 | 0.7812 | 0.7114 | 0.7442 | 0.6443 | 0.6906 |
| 2 | 0 | 0 | 1 | 0.7342 | 0.6107 | 0.7260 | 0.3557 | 0.4775 |
| 2 | 0 | 0 | 2 | 0.6624 | 0.6779 | 0.6934 | 0.6376 | 0.6643 |
| 2 | 0 | 0 | 3 | 0.6858 | 0.5000 | 0.0000 | 0.0000 | 0.0000 |
| 2 | 0 | 0 | 4 | 0.6193 | 0.5336 | 0.8125 | 0.0872 | 0.1576 |
| 2 | 0 | 0 | 5 | 0.7304 | 0.6812 | 0.7935 | 0.4899 | 0.6058 |
| 2 | 0 | 0 | 6 | 0.7234 | 0.5638 | 0.9130 | 0.1409 | 0.2442 |
| 2 | 0 | 0 | 7 | 0.7125 | 0.5201 | 1.0000 | 0.0403 | 0.0774 |
| 2 | 0 | 0 | 8 | 0.6439 | 0.5436 | 0.7600 | 0.1275 | 0.2184 |
| 2 | 0 | 1 | 0 | 0.6563 | 0.5973 | 0.6381 | 0.4497 | 0.5276 |
| 2 | 0 | 1 | 1 | 0.7042 | 0.6611 | 0.7400 | 0.4966 | 0.5944 |
| 2 | 0 | 1 | 2 | 0.6718 | 0.5973 | 0.6381 | 0.4497 | 0.5276 |
| 2 | 0 | 1 | 3 | 0.6633 | 0.5403 | 1.0000 | 0.0805 | 0.1491 |
| 2 | 0 | 1 | 4 | 0.5343 | 0.5671 | 0.8125 | 0.1745 | 0.2873 |
| 2 | 0 | 1 | 5 | 0.5559 | 0.5906 | 0.6552 | 0.3826 | 0.4831 |
| 2 | 0 | 1 | 6 | 0.6266 | 0.5705 | 0.8387 | 0.1745 | 0.2889 |
| 2 | 0 | 1 | 7 | 0.5773 | 0.5570 | 0.9048 | 0.1275 | 0.2235 |
| 2 | 0 | 1 | 8 | 0.6192 | 0.5671 | 0.7000 | 0.2349 | 0.3518 |
| 2 | 0 | 2 | 0 | 0.5923 | 0.5537 | 0.5580 | 0.5168 | 0.5366 |
| 2 | 0 | 2 | 1 | 0.4935 | 0.4966 | 0.4958 | 0.3960 | 0.4403 |
| 2 | 0 | 2 | 2 | 0.5304 | 0.4866 | 0.4444 | 0.1074 | 0.1730 |
| 2 | 0 | 2 | 3 | 0.5004 | 0.4396 | 0.3784 | 0.1879 | 0.2511 |
| 2 | 0 | 2 | 4 | 0.5300 | 0.4497 | 0.4026 | 0.2081 | 0.2743 |
| 2 | 0 | 2 | 5 | 0.5952 | 0.5537 | 0.5580 | 0.5168 | 0.5366 |
| 2 | 0 | 2 | 6 | 0.5027 | 0.4899 | 0.4831 | 0.2886 | 0.3613 |
| 2 | 0 | 2 | 7 | 0.5327 | 0.4799 | 0.4706 | 0.3221 | 0.3825 |
| 2 | 0 | 2 | 8 | 0.5560 | 0.5336 | 0.5385 | 0.4698 | 0.5018 |
| 2 | 0 | 3 | 0 | 0.7812 | 0.7114 | 0.7442 | 0.6443 | 0.6906 |
| 2 | 0 | 3 | 1 | 0.7342 | 0.6107 | 0.7260 | 0.3557 | 0.4775 |
| 2 | 0 | 3 | 2 | 0.6624 | 0.6779 | 0.6934 | 0.6376 | 0.6643 |
| 2 | 0 | 3 | 3 | 0.6858 | 0.5000 | 0.0000 | 0.0000 | 0.0000 |
| 2 | 0 | 3 | 4 | 0.6193 | 0.5336 | 0.8125 | 0.0872 | 0.1576 |
| 2 | 0 | 3 | 5 | 0.7304 | 0.6812 | 0.7935 | 0.4899 | 0.6058 |
| 2 | 0 | 3 | 6 | 0.7234 | 0.5638 | 0.9130 | 0.1409 | 0.2442 |
| 2 | 0 | 3 | 7 | 0.7125 | 0.5201 | 1.0000 | 0.0403 | 0.0774 |
| 2 | 0 | 3 | 8 | 0.6439 | 0.5436 | 0.7600 | 0.1275 | 0.2184 |
| 2 | 1 | 0 | 0 | 0.7619 | 0.6644 | 0.7059 | 0.5638 | 0.6269 |
| 2 | 1 | 0 | 1 | 0.7787 | 0.6913 | 0.7767 | 0.5369 | 0.6349 |
| 2 | 1 | 0 | 2 | 0.7058 | 0.6711 | 0.6378 | 0.7919 | 0.7066 |
| 2 | 1 | 0 | 3 | 0.9469 | 0.7919 | 0.9780 | 0.5973 | 0.7417 |
| 2 | 1 | 0 | 4 | 0.9451 | 0.8792 | 0.9913 | 0.7651 | 0.8636 |
| 2 | 1 | 0 | 5 | 0.9498 | 0.8893 | 0.9265 | 0.8456 | 0.8842 |
| 2 | 1 | 0 | 6 | 0.9395 | 0.8456 | 0.9905 | 0.6980 | 0.8189 |
| 2 | 1 | 0 | 7 | 0.9568 | 0.8691 | 0.9911 | 0.7450 | 0.8506 |
| 2 | 1 | 0 | 8 | 0.9564 | 0.8960 | 0.9836 | 0.8054 | 0.8856 |
| 2 | 1 | 1 | 0 | 0.7589 | 0.6309 | 0.6696 | 0.5168 | 0.5833 |
| 2 | 1 | 1 | 1 | 0.7930 | 0.7248 | 0.7638 | 0.6510 | 0.7029 |
| 2 | 1 | 1 | 2 | 0.6883 | 0.6242 | 0.6225 | 0.6309 | 0.6267 |
| 2 | 1 | 1 | 3 | 0.9524 | 0.8557 | 0.9732 | 0.7315 | 0.8352 |
| 2 | 1 | 1 | 4 | 0.9441 | 0.8691 | 0.9583 | 0.7718 | 0.8550 |
| 2 | 1 | 1 | 5 | 0.9462 | 0.8960 | 0.9155 | 0.8725 | 0.8935 |
| 2 | 1 | 1 | 6 | 0.9461 | 0.8792 | 0.9748 | 0.7785 | 0.8657 |
| 2 | 1 | 1 | 7 | 0.9478 | 0.8926 | 0.9680 | 0.8121 | 0.8832 |
| 2 | 1 | 1 | 8 | 0.9582 | 0.9195 | 0.9771 | 0.8591 | 0.9143 |
| 2 | 1 | 2 | 0 | 0.4673 | 0.4329 | 0.4485 | 0.5839 | 0.5073 |
| 2 | 1 | 2 | 1 | 0.6279 | 0.5403 | 0.5309 | 0.6913 | 0.6006 |
| 2 | 1 | 2 | 2 | 0.7732 | 0.7517 | 0.9412 | 0.5369 | 0.6838 |
| 2 | 1 | 2 | 3 | 0.7715 | 0.7483 | 0.8776 | 0.5772 | 0.6964 |
| 2 | 1 | 2 | 4 | 0.8097 | 0.7785 | 0.9029 | 0.6242 | 0.7381 |
| 2 | 1 | 2 | 5 | 0.7544 | 0.7685 | 0.9762 | 0.5503 | 0.7039 |
| 2 | 1 | 2 | 6 | 0.8005 | 0.7752 | 0.9020 | 0.6174 | 0.7331 |
| 2 | 1 | 2 | 7 | 0.7869 | 0.7685 | 0.9167 | 0.5906 | 0.7184 |
| 2 | 1 | 2 | 8 | 0.7924 | 0.7651 | 0.9158 | 0.5839 | 0.7131 |
| 2 | 1 | 3 | 0 | 0.7619 | 0.6644 | 0.7059 | 0.5638 | 0.6269 |
| 2 | 1 | 3 | 1 | 0.7787 | 0.6913 | 0.7767 | 0.5369 | 0.6349 |
| 2 | 1 | 3 | 2 | 0.7058 | 0.6711 | 0.6378 | 0.7919 | 0.7066 |
| 2 | 1 | 3 | 3 | 0.9469 | 0.7919 | 0.9780 | 0.5973 | 0.7417 |
| 2 | 1 | 3 | 4 | 0.9451 | 0.8792 | 0.9913 | 0.7651 | 0.8636 |
| 2 | 1 | 3 | 5 | 0.9498 | 0.8893 | 0.9265 | 0.8456 | 0.8842 |
| 2 | 1 | 3 | 6 | 0.9395 | 0.8456 | 0.9905 | 0.6980 | 0.8189 |
| 2 | 1 | 3 | 7 | 0.9568 | 0.8691 | 0.9911 | 0.7450 | 0.8506 |
| 2 | 1 | 3 | 8 | 0.9564 | 0.8960 | 0.9836 | 0.8054 | 0.8856 |
| 2 | 2 | 0 | 0 | 0.7891 | 0.6678 | 0.7273 | 0.5369 | 0.6178 |
| 2 | 2 | 0 | 1 | 0.7746 | 0.6779 | 0.7732 | 0.5034 | 0.6098 |
| 2 | 2 | 0 | 2 | 0.6827 | 0.6544 | 0.6369 | 0.7181 | 0.6751 |
| 2 | 2 | 0 | 3 | 0.9455 | 0.7987 | 0.9890 | 0.6040 | 0.7500 |
| 2 | 2 | 0 | 4 | 0.9288 | 0.8154 | 0.9608 | 0.6577 | 0.7809 |
| 2 | 2 | 0 | 5 | 0.9334 | 0.8591 | 0.9023 | 0.8054 | 0.8511 |
| 2 | 2 | 0 | 6 | 0.9332 | 0.8087 | 1.0000 | 0.6174 | 0.7635 |
| 2 | 2 | 0 | 7 | 0.9412 | 0.8087 | 0.9894 | 0.6242 | 0.7654 |
| 2 | 2 | 0 | 8 | 0.9489 | 0.8691 | 0.9741 | 0.7584 | 0.8528 |
| 2 | 2 | 1 | 0 | 0.7735 | 0.6074 | 0.6429 | 0.4832 | 0.5517 |
| 2 | 2 | 1 | 1 | 0.8028 | 0.7383 | 0.7934 | 0.6443 | 0.7111 |
| 2 | 2 | 1 | 2 | 0.7271 | 0.6711 | 0.6711 | 0.6711 | 0.6711 |
| 2 | 2 | 1 | 3 | 0.9350 | 0.8356 | 0.9902 | 0.6779 | 0.8048 |
| 2 | 2 | 1 | 4 | 0.9313 | 0.8591 | 0.9908 | 0.7248 | 0.8372 |
| 2 | 2 | 1 | 5 | 0.8978 | 0.8624 | 0.9426 | 0.7718 | 0.8487 |
| 2 | 2 | 1 | 6 | 0.9383 | 0.8523 | 0.9339 | 0.7584 | 0.8370 |
| 2 | 2 | 1 | 7 | 0.9330 | 0.8490 | 0.9815 | 0.7114 | 0.8249 |
| 2 | 2 | 1 | 8 | 0.9357 | 0.8658 | 0.9431 | 0.7785 | 0.8529 |
| 2 | 2 | 2 | 0 | 0.3396 | 0.3691 | 0.3673 | 0.3624 | 0.3649 |
| 2 | 2 | 2 | 1 | 0.5242 | 0.5067 | 0.5059 | 0.5772 | 0.5392 |
| 2 | 2 | 2 | 2 | 0.7934 | 0.7416 | 0.9091 | 0.5369 | 0.6751 |
| 2 | 2 | 2 | 3 | 0.7624 | 0.6007 | 0.5765 | 0.7584 | 0.6551 |
| 2 | 2 | 2 | 4 | 0.7785 | 0.6040 | 0.5779 | 0.7718 | 0.6609 |
| 2 | 2 | 2 | 5 | 0.7719 | 0.6074 | 0.5800 | 0.7785 | 0.6648 |
| 2 | 2 | 2 | 6 | 0.7427 | 0.5772 | 0.5590 | 0.7315 | 0.6337 |
| 2 | 2 | 2 | 7 | 0.7671 | 0.5940 | 0.5707 | 0.7584 | 0.6513 |
| 2 | 2 | 2 | 8 | 0.7679 | 0.5872 | 0.5663 | 0.7450 | 0.6435 |
| 2 | 2 | 3 | 0 | 0.7891 | 0.6678 | 0.7273 | 0.5369 | 0.6178 |
| 2 | 2 | 3 | 1 | 0.7746 | 0.6779 | 0.7732 | 0.5034 | 0.6098 |
| 2 | 2 | 3 | 2 | 0.6827 | 0.6544 | 0.6369 | 0.7181 | 0.6751 |
| 2 | 2 | 3 | 3 | 0.9455 | 0.7987 | 0.9890 | 0.6040 | 0.7500 |
| 2 | 2 | 3 | 4 | 0.9288 | 0.8154 | 0.9608 | 0.6577 | 0.7809 |
| 2 | 2 | 3 | 5 | 0.9334 | 0.8591 | 0.9023 | 0.8054 | 0.8511 |
| 2 | 2 | 3 | 6 | 0.9332 | 0.8087 | 1.0000 | 0.6174 | 0.7635 |
| 2 | 2 | 3 | 7 | 0.9412 | 0.8087 | 0.9894 | 0.6242 | 0.7654 |
| 2 | 2 | 3 | 8 | 0.9489 | 0.8691 | 0.9741 | 0.7584 | 0.8528 |
| 2 | 3 | 0 | 0 | 0.9443 | 0.8993 | 0.9281 | 0.8658 | 0.8958 |
| 2 | 3 | 0 | 1 | 0.9467 | 0.8792 | 0.9829 | 0.7718 | 0.8647 |
| 2 | 3 | 0 | 2 | 0.8810 | 0.8423 | 0.8806 | 0.7919 | 0.8339 |
| 2 | 3 | 0 | 3 | 0.9455 | 0.9195 | 1.0000 | 0.8389 | 0.9124 |
| 2 | 3 | 0 | 4 | 0.9382 | 0.8893 | 0.9754 | 0.7987 | 0.8782 |
| 2 | 3 | 0 | 5 | 0.9016 | 0.8221 | 0.9138 | 0.7114 | 0.8000 |
| 2 | 3 | 0 | 6 | 0.9449 | 0.8960 | 0.9836 | 0.8054 | 0.8856 |
| 2 | 3 | 0 | 7 | 0.9468 | 0.9161 | 0.9921 | 0.8389 | 0.9091 |
| 2 | 3 | 0 | 8 | 0.9405 | 0.8960 | 0.9683 | 0.8188 | 0.8873 |
| 2 | 3 | 1 | 0 | 0.9338 | 0.8993 | 0.9220 | 0.8725 | 0.8966 |
| 2 | 3 | 1 | 1 | 0.9330 | 0.8557 | 0.9732 | 0.7315 | 0.8352 |
| 2 | 3 | 1 | 2 | 0.8578 | 0.8020 | 0.8462 | 0.7383 | 0.7885 |
| 2 | 3 | 1 | 3 | 0.9379 | 0.8893 | 0.9833 | 0.7919 | 0.8773 |
| 2 | 3 | 1 | 4 | 0.9283 | 0.8591 | 0.9573 | 0.7517 | 0.8421 |
| 2 | 3 | 1 | 5 | 0.7311 | 0.7584 | 0.8235 | 0.6577 | 0.7313 |
| 2 | 3 | 1 | 6 | 0.9219 | 0.8289 | 0.9455 | 0.6980 | 0.8031 |
| 2 | 3 | 1 | 7 | 0.9330 | 0.9161 | 0.9844 | 0.8456 | 0.9097 |
| 2 | 3 | 1 | 8 | 0.9329 | 0.8993 | 0.9612 | 0.8322 | 0.8921 |
| 2 | 3 | 2 | 0 | 0.5832 | 0.4933 | 0.4894 | 0.3087 | 0.3786 |
| 2 | 3 | 2 | 1 | 0.6080 | 0.5403 | 0.5588 | 0.3826 | 0.4542 |
| 2 | 3 | 2 | 2 | 0.6102 | 0.5403 | 0.7000 | 0.1409 | 0.2346 |
| 2 | 3 | 2 | 3 | 0.5582 | 0.5872 | 0.6048 | 0.5034 | 0.5495 |
| 2 | 3 | 2 | 4 | 0.6060 | 0.6007 | 0.6210 | 0.5168 | 0.5641 |
| 2 | 3 | 2 | 5 | 0.5997 | 0.5503 | 0.5600 | 0.4698 | 0.5109 |
| 2 | 3 | 2 | 6 | 0.5882 | 0.5872 | 0.6121 | 0.4765 | 0.5358 |
| 2 | 3 | 2 | 7 | 0.5997 | 0.6107 | 0.6387 | 0.5101 | 0.5672 |
| 2 | 3 | 2 | 8 | 0.6271 | 0.6007 | 0.6103 | 0.5570 | 0.5825 |
| 2 | 3 | 3 | 0 | 0.9443 | 0.8993 | 0.9281 | 0.8658 | 0.8958 |
| 2 | 3 | 3 | 1 | 0.9467 | 0.8792 | 0.9829 | 0.7718 | 0.8647 |
| 2 | 3 | 3 | 2 | 0.8810 | 0.8423 | 0.8806 | 0.7919 | 0.8339 |
| 2 | 3 | 3 | 3 | 0.9455 | 0.9195 | 1.0000 | 0.8389 | 0.9124 |
| 2 | 3 | 3 | 4 | 0.9382 | 0.8893 | 0.9754 | 0.7987 | 0.8782 |
| 2 | 3 | 3 | 5 | 0.9016 | 0.8221 | 0.9138 | 0.7114 | 0.8000 |
| 2 | 3 | 3 | 6 | 0.9449 | 0.8960 | 0.9836 | 0.8054 | 0.8856 |
| 2 | 3 | 3 | 7 | 0.9468 | 0.9161 | 0.9921 | 0.8389 | 0.9091 |
| 2 | 3 | 3 | 8 | 0.9405 | 0.8960 | 0.9683 | 0.8188 | 0.8873 |
| 3 | 0 | 0 | 0 | 0.6619 | 0.6049 | 0.6596 | 0.4336 | 0.5232 |
| 3 | 0 | 0 | 1 | 0.6787 | 0.5559 | 0.6333 | 0.2657 | 0.3744 |
| 3 | 0 | 0 | 2 | 0.4812 | 0.4965 | 0.4928 | 0.2378 | 0.3208 |
| 3 | 0 | 0 | 3 | 0.6439 | 0.4965 | 0.0000 | 0.0000 | 0.0000 |
| 3 | 0 | 0 | 4 | 0.6794 | 0.5385 | 0.7619 | 0.1119 | 0.1951 |
| 3 | 0 | 0 | 5 | 0.6632 | 0.6224 | 0.7059 | 0.4196 | 0.5263 |
| 3 | 0 | 0 | 6 | 0.6877 | 0.5629 | 0.8462 | 0.1538 | 0.2604 |
| 3 | 0 | 0 | 7 | 0.6892 | 0.5175 | 0.7273 | 0.0559 | 0.1039 |
| 3 | 0 | 0 | 8 | 0.6838 | 0.5315 | 0.6047 | 0.1818 | 0.2796 |
| 3 | 0 | 1 | 0 | 0.6949 | 0.6469 | 0.6981 | 0.5175 | 0.5944 |
| 3 | 0 | 1 | 1 | 0.6567 | 0.5699 | 0.6316 | 0.3357 | 0.4384 |
| 3 | 0 | 1 | 2 | 0.4057 | 0.4336 | 0.3333 | 0.1329 | 0.1900 |
| 3 | 0 | 1 | 3 | 0.6991 | 0.4895 | 0.0000 | 0.0000 | 0.0000 |
| 3 | 0 | 1 | 4 | 0.6222 | 0.4965 | 0.4615 | 0.0420 | 0.0769 |
| 3 | 0 | 1 | 5 | 0.6419 | 0.5944 | 0.6753 | 0.3636 | 0.4727 |
| 3 | 0 | 1 | 6 | 0.6452 | 0.5210 | 0.6875 | 0.0769 | 0.1384 |
| 3 | 0 | 1 | 7 | 0.6903 | 0.5070 | 0.6000 | 0.0420 | 0.0784 |
| 3 | 0 | 1 | 8 | 0.6551 | 0.4860 | 0.4333 | 0.0909 | 0.1503 |
| 3 | 0 | 2 | 0 | 0.6834 | 0.6154 | 0.6204 | 0.5944 | 0.6071 |
| 3 | 0 | 2 | 1 | 0.6257 | 0.5664 | 0.5979 | 0.4056 | 0.4833 |
| 3 | 0 | 2 | 2 | 0.6331 | 0.5594 | 0.6604 | 0.2448 | 0.3571 |
| 3 | 0 | 2 | 3 | 0.5897 | 0.5245 | 0.5385 | 0.3427 | 0.4188 |
| 3 | 0 | 2 | 4 | 0.5400 | 0.4965 | 0.4932 | 0.2517 | 0.3333 |
| 3 | 0 | 2 | 5 | 0.6904 | 0.6224 | 0.6296 | 0.5944 | 0.6115 |
| 3 | 0 | 2 | 6 | 0.5837 | 0.5175 | 0.5301 | 0.3077 | 0.3894 |
| 3 | 0 | 2 | 7 | 0.5242 | 0.4930 | 0.4865 | 0.2517 | 0.3318 |
| 3 | 0 | 2 | 8 | 0.5152 | 0.5035 | 0.5054 | 0.3287 | 0.3983 |
| 3 | 0 | 3 | 0 | 0.6619 | 0.6049 | 0.6596 | 0.4336 | 0.5232 |
| 3 | 0 | 3 | 1 | 0.6787 | 0.5559 | 0.6333 | 0.2657 | 0.3744 |
| 3 | 0 | 3 | 2 | 0.4812 | 0.4965 | 0.4928 | 0.2378 | 0.3208 |
| 3 | 0 | 3 | 3 | 0.6439 | 0.4965 | 0.0000 | 0.0000 | 0.0000 |
| 3 | 0 | 3 | 4 | 0.6794 | 0.5385 | 0.7619 | 0.1119 | 0.1951 |
| 3 | 0 | 3 | 5 | 0.6632 | 0.6224 | 0.7059 | 0.4196 | 0.5263 |
| 3 | 0 | 3 | 6 | 0.6877 | 0.5629 | 0.8462 | 0.1538 | 0.2604 |
| 3 | 0 | 3 | 7 | 0.6892 | 0.5175 | 0.7273 | 0.0559 | 0.1039 |
| 3 | 0 | 3 | 8 | 0.6838 | 0.5315 | 0.6047 | 0.1818 | 0.2796 |
| 3 | 1 | 0 | 0 | 0.8998 | 0.8462 | 0.8837 | 0.7972 | 0.8382 |
| 3 | 1 | 0 | 1 | 0.8619 | 0.7552 | 0.8687 | 0.6014 | 0.7107 |
| 3 | 1 | 0 | 2 | 0.6542 | 0.6014 | 0.6014 | 0.6014 | 0.6014 |
| 3 | 1 | 0 | 3 | 0.8991 | 0.7343 | 0.9467 | 0.4965 | 0.6514 |
| 3 | 1 | 0 | 4 | 0.9099 | 0.8147 | 0.9500 | 0.6643 | 0.7819 |
| 3 | 1 | 0 | 5 | 0.8463 | 0.8007 | 0.8707 | 0.7063 | 0.7799 |
| 3 | 1 | 0 | 6 | 0.8931 | 0.7587 | 0.9302 | 0.5594 | 0.6987 |
| 3 | 1 | 0 | 7 | 0.9134 | 0.7972 | 0.9474 | 0.6294 | 0.7563 |
| 3 | 1 | 0 | 8 | 0.9077 | 0.8147 | 0.9167 | 0.6923 | 0.7888 |
| 3 | 1 | 1 | 0 | 0.8990 | 0.8531 | 0.8915 | 0.8042 | 0.8456 |
| 3 | 1 | 1 | 1 | 0.8659 | 0.7552 | 0.8763 | 0.5944 | 0.7083 |
| 3 | 1 | 1 | 2 | 0.7618 | 0.6923 | 0.7068 | 0.6573 | 0.6812 |
| 3 | 1 | 1 | 3 | 0.8901 | 0.7657 | 0.9222 | 0.5804 | 0.7124 |
| 3 | 1 | 1 | 4 | 0.9180 | 0.8217 | 0.9259 | 0.6993 | 0.7968 |
| 3 | 1 | 1 | 5 | 0.8969 | 0.8357 | 0.8380 | 0.8322 | 0.8351 |
| 3 | 1 | 1 | 6 | 0.9233 | 0.8427 | 0.9298 | 0.7413 | 0.8249 |
| 3 | 1 | 1 | 7 | 0.9081 | 0.8287 | 0.9273 | 0.7133 | 0.8063 |
| 3 | 1 | 1 | 8 | 0.9127 | 0.8322 | 0.8992 | 0.7483 | 0.8168 |
| 3 | 1 | 2 | 0 | 0.6262 | 0.6014 | 0.6000 | 0.6084 | 0.6042 |
| 3 | 1 | 2 | 1 | 0.5377 | 0.5175 | 0.5248 | 0.3706 | 0.4344 |
| 3 | 1 | 2 | 2 | 0.5122 | 0.5420 | 0.7308 | 0.1329 | 0.2249 |
| 3 | 1 | 2 | 3 | 0.5532 | 0.5280 | 0.5465 | 0.3287 | 0.4105 |
| 3 | 1 | 2 | 4 | 0.5771 | 0.5210 | 0.5306 | 0.3636 | 0.4315 |
| 3 | 1 | 2 | 5 | 0.6311 | 0.6154 | 0.6107 | 0.6364 | 0.6233 |
| 3 | 1 | 2 | 6 | 0.5622 | 0.5035 | 0.5051 | 0.3497 | 0.4132 |
| 3 | 1 | 2 | 7 | 0.5648 | 0.5175 | 0.5275 | 0.3357 | 0.4103 |
| 3 | 1 | 2 | 8 | 0.5495 | 0.5280 | 0.5351 | 0.4266 | 0.4747 |
| 3 | 1 | 3 | 0 | 0.8998 | 0.8462 | 0.8837 | 0.7972 | 0.8382 |
| 3 | 1 | 3 | 1 | 0.8619 | 0.7552 | 0.8687 | 0.6014 | 0.7107 |
| 3 | 1 | 3 | 2 | 0.6542 | 0.6014 | 0.6014 | 0.6014 | 0.6014 |
| 3 | 1 | 3 | 3 | 0.8991 | 0.7343 | 0.9467 | 0.4965 | 0.6514 |
| 3 | 1 | 3 | 4 | 0.9099 | 0.8147 | 0.9500 | 0.6643 | 0.7819 |
| 3 | 1 | 3 | 5 | 0.8463 | 0.8007 | 0.8707 | 0.7063 | 0.7799 |
| 3 | 1 | 3 | 6 | 0.8931 | 0.7587 | 0.9302 | 0.5594 | 0.6987 |
| 3 | 1 | 3 | 7 | 0.9134 | 0.7972 | 0.9474 | 0.6294 | 0.7563 |
| 3 | 1 | 3 | 8 | 0.9077 | 0.8147 | 0.9167 | 0.6923 | 0.7888 |
| 3 | 2 | 0 | 0 | 0.8829 | 0.8077 | 0.8492 | 0.7483 | 0.7955 |
| 3 | 2 | 0 | 1 | 0.8521 | 0.7343 | 0.8764 | 0.5455 | 0.6724 |
| 3 | 2 | 0 | 2 | 0.6468 | 0.5944 | 0.6080 | 0.5315 | 0.5672 |
| 3 | 2 | 0 | 3 | 0.8698 | 0.6503 | 0.9574 | 0.3147 | 0.4737 |
| 3 | 2 | 0 | 4 | 0.8828 | 0.7378 | 0.9048 | 0.5315 | 0.6696 |
| 3 | 2 | 0 | 5 | 0.8677 | 0.8147 | 0.8689 | 0.7413 | 0.8000 |
| 3 | 2 | 0 | 6 | 0.8903 | 0.7273 | 0.9221 | 0.4965 | 0.6455 |
| 3 | 2 | 0 | 7 | 0.9013 | 0.7622 | 0.9412 | 0.5594 | 0.7018 |
| 3 | 2 | 0 | 8 | 0.8915 | 0.7797 | 0.9082 | 0.6224 | 0.7386 |
| 3 | 2 | 1 | 0 | 0.8830 | 0.8252 | 0.8605 | 0.7762 | 0.8162 |
| 3 | 2 | 1 | 1 | 0.8551 | 0.7063 | 0.8242 | 0.5245 | 0.6410 |
| 3 | 2 | 1 | 2 | 0.7279 | 0.6853 | 0.7154 | 0.6154 | 0.6617 |
| 3 | 2 | 1 | 3 | 0.8988 | 0.7622 | 0.9412 | 0.5594 | 0.7018 |
| 3 | 2 | 1 | 4 | 0.9055 | 0.7832 | 0.9175 | 0.6224 | 0.7417 |
| 3 | 2 | 1 | 5 | 0.8977 | 0.8427 | 0.8551 | 0.8252 | 0.8399 |
| 3 | 2 | 1 | 6 | 0.9238 | 0.8322 | 0.9358 | 0.7133 | 0.8095 |
| 3 | 2 | 1 | 7 | 0.9061 | 0.7902 | 0.9462 | 0.6154 | 0.7458 |
| 3 | 2 | 1 | 8 | 0.9050 | 0.8392 | 0.9217 | 0.7413 | 0.8217 |
| 3 | 2 | 2 | 0 | 0.5811 | 0.6119 | 0.5899 | 0.7343 | 0.6542 |
| 3 | 2 | 2 | 1 | 0.6149 | 0.5559 | 0.5714 | 0.4476 | 0.5020 |
| 3 | 2 | 2 | 2 | 0.4896 | 0.5035 | 0.5217 | 0.0839 | 0.1446 |
| 3 | 2 | 2 | 3 | 0.5679 | 0.4650 | 0.4432 | 0.2727 | 0.3377 |
| 3 | 2 | 2 | 4 | 0.6196 | 0.5594 | 0.5794 | 0.4336 | 0.4960 |
| 3 | 2 | 2 | 5 | 0.5787 | 0.5909 | 0.5707 | 0.7343 | 0.6422 |
| 3 | 2 | 2 | 6 | 0.6029 | 0.5315 | 0.5405 | 0.4196 | 0.4724 |
| 3 | 2 | 2 | 7 | 0.6041 | 0.5455 | 0.5631 | 0.4056 | 0.4715 |
| 3 | 2 | 2 | 8 | 0.6296 | 0.5524 | 0.5630 | 0.4685 | 0.5115 |
| 3 | 2 | 3 | 0 | 0.8829 | 0.8077 | 0.8492 | 0.7483 | 0.7955 |
| 3 | 2 | 3 | 1 | 0.8521 | 0.7343 | 0.8764 | 0.5455 | 0.6724 |
| 3 | 2 | 3 | 2 | 0.6468 | 0.5944 | 0.6080 | 0.5315 | 0.5672 |
| 3 | 2 | 3 | 3 | 0.8698 | 0.6503 | 0.9574 | 0.3147 | 0.4737 |
| 3 | 2 | 3 | 4 | 0.8828 | 0.7378 | 0.9048 | 0.5315 | 0.6696 |
| 3 | 2 | 3 | 5 | 0.8677 | 0.8147 | 0.8689 | 0.7413 | 0.8000 |
| 3 | 2 | 3 | 6 | 0.8903 | 0.7273 | 0.9221 | 0.4965 | 0.6455 |
| 3 | 2 | 3 | 7 | 0.9013 | 0.7622 | 0.9412 | 0.5594 | 0.7018 |
| 3 | 2 | 3 | 8 | 0.8915 | 0.7797 | 0.9082 | 0.6224 | 0.7386 |
| 3 | 3 | 0 | 0 | 0.8852 | 0.8741 | 0.9280 | 0.8112 | 0.8657 |
| 3 | 3 | 0 | 1 | 0.9198 | 0.8881 | 0.9826 | 0.7902 | 0.8760 |
| 3 | 3 | 0 | 2 | 0.8822 | 0.8566 | 0.8806 | 0.8252 | 0.8520 |
| 3 | 3 | 0 | 3 | 0.9179 | 0.8811 | 1.0000 | 0.7622 | 0.8651 |
| 3 | 3 | 0 | 4 | 0.9096 | 0.8182 | 0.9596 | 0.6643 | 0.7851 |
| 3 | 3 | 0 | 5 | 0.7939 | 0.6748 | 0.8289 | 0.4406 | 0.5753 |
| 3 | 3 | 0 | 6 | 0.9124 | 0.8497 | 0.9630 | 0.7273 | 0.8287 |
| 3 | 3 | 0 | 7 | 0.9252 | 0.9056 | 1.0000 | 0.8112 | 0.8958 |
| 3 | 3 | 0 | 8 | 0.9106 | 0.8462 | 0.9381 | 0.7413 | 0.8281 |
| 3 | 3 | 1 | 0 | 0.8880 | 0.8916 | 0.9590 | 0.8182 | 0.8830 |
| 3 | 3 | 1 | 1 | 0.9341 | 0.8916 | 0.9746 | 0.8042 | 0.8812 |
| 3 | 3 | 1 | 2 | 0.8980 | 0.8846 | 0.9365 | 0.8252 | 0.8773 |
| 3 | 3 | 1 | 3 | 0.9274 | 0.8776 | 1.0000 | 0.7552 | 0.8606 |
| 3 | 3 | 1 | 4 | 0.9123 | 0.8706 | 0.9417 | 0.7902 | 0.8593 |
| 3 | 3 | 1 | 5 | 0.8536 | 0.7343 | 0.8764 | 0.5455 | 0.6724 |
| 3 | 3 | 1 | 6 | 0.9194 | 0.8497 | 0.9545 | 0.7343 | 0.8300 |
| 3 | 3 | 1 | 7 | 0.9284 | 0.8951 | 0.9829 | 0.8042 | 0.8846 |
| 3 | 3 | 1 | 8 | 0.9119 | 0.8531 | 0.9550 | 0.7413 | 0.8346 |
| 3 | 3 | 2 | 0 | 0.6414 | 0.5664 | 0.5888 | 0.4406 | 0.5040 |
| 3 | 3 | 2 | 1 | 0.7280 | 0.6888 | 0.7177 | 0.6224 | 0.6667 |
| 3 | 3 | 2 | 2 | 0.5873 | 0.5804 | 0.7949 | 0.2168 | 0.3407 |
| 3 | 3 | 2 | 3 | 0.6625 | 0.6399 | 0.6887 | 0.5105 | 0.5863 |
| 3 | 3 | 2 | 4 | 0.6988 | 0.6783 | 0.7179 | 0.5874 | 0.6462 |
| 3 | 3 | 2 | 5 | 0.6236 | 0.5524 | 0.5676 | 0.4406 | 0.4961 |
| 3 | 3 | 2 | 6 | 0.7131 | 0.6538 | 0.6964 | 0.5455 | 0.6118 |
| 3 | 3 | 2 | 7 | 0.7107 | 0.6469 | 0.6875 | 0.5385 | 0.6039 |
| 3 | 3 | 2 | 8 | 0.7090 | 0.6923 | 0.7099 | 0.6503 | 0.6788 |
| 3 | 3 | 3 | 0 | 0.8852 | 0.8741 | 0.9280 | 0.8112 | 0.8657 |
| 3 | 3 | 3 | 1 | 0.9198 | 0.8881 | 0.9826 | 0.7902 | 0.8760 |
| 3 | 3 | 3 | 2 | 0.8822 | 0.8566 | 0.8806 | 0.8252 | 0.8520 |
| 3 | 3 | 3 | 3 | 0.9179 | 0.8811 | 1.0000 | 0.7622 | 0.8651 |
| 3 | 3 | 3 | 4 | 0.9096 | 0.8182 | 0.9596 | 0.6643 | 0.7851 |
| 3 | 3 | 3 | 5 | 0.7939 | 0.6748 | 0.8289 | 0.4406 | 0.5753 |
| 3 | 3 | 3 | 6 | 0.9124 | 0.8497 | 0.9630 | 0.7273 | 0.8287 |
| 3 | 3 | 3 | 7 | 0.9252 | 0.9056 | 1.0000 | 0.8112 | 0.8958 |
| 3 | 3 | 3 | 8 | 0.9106 | 0.8462 | 0.9381 | 0.7413 | 0.8281 |
